# Influence of ZnO Addition on the Structural and Biological Properties of Borate Bioactive Glasses for Bone Tissue Regeneration

**DOI:** 10.1021/acsomega.6c05452

**Published:** 2026-08-16

**Authors:** Taís Silva Morais, Otávio Cândido da Silva Neto, Maria Nayane de Queiroz, Tatielle Gomes Dias, Fernando Liberalino Fernandes, Daria Raquel Queiroz de Almeida, Tatiana Mesquita Basto Maia, Aramys Silva Reis, Richard Pereira Dutra, Thales Allyrio Araújo de Medeiros Fernandes, Alysson Steimacher, Franciana Pedrochi

**Affiliations:** † Programa de Pós-graduação em Ciência dos Materiais, Centro de Ciências de Imperatriz, Universidade Federal do Maranhão, 65915-240 Imperatriz, MA, Brazil; ‡ Programa de Pós-graduação em Saúde e Tecnologia, Centro de Ciências de Imperatriz, Universidade Federal do Maranhão, 65915-240 Imperatriz, MA, Brazil; § 125287 Universidade Ceuma, Campus Imperatriz, Rua Barão do Rio Branco, n° 100, 65903-093 Imperatriz, Ma, Brazil; ∥ Faculdade de Ciências da Saúde—FACS, Universidade do Estado do Rio Grande do Norte—UERN, 59607-360 Mossoró, RN, Brazil

## Abstract

This research evaluated the ZnO influence on the bioactivity of the vitreous system formulated as ((64-x) B_2_O_3_–2P_2_O_5_–18 CaO–16Na_2_O–xZnO, with *x* = 0, 1, 2.5, 5, 7.5, and 10 mol %). The glasses were synthesized by melt-quenching technique and characterized by density, molar volume, FTIR, *N*_4_, XRD, radiopacity, *in vitro* test in SBF, mass loss, SEM-EDS cellular viability, cell migration assay, and antibacterial activity. The volumetric density and N_4_ increased, and the molar volume decreased, indicating that the addition of ZnO changed the structure of the materials. DTA analysis showed that *T*_g_ did not vary significantly, *T*_x_ increased due to the ZnO addition, and all samples presented good thermal stability. The radiopacity level of the glass samples was enhanced with ZnO addition. Also, analyses of mass loss and pH measurements shown an increase in the chemical stability of samples with ZnO contents. After immersion in SBF, the XRD diffractograms and FTIR spectra of glasses indicated the bioactivity of the CaNaB, 2.5% ZnO, and 5% ZnO samples by the presence of peaks and bands characteristic of HA formation. By cell viability analysis using RAW-264.7 murine macrophages, all glasses were shown to be noncytotoxic. Bioactive glasses compositions preserve the intrinsic migratory capacity of human fibroblasts. All glass samples showed antibacterial activity against Gram-positive and Gram-negative bacteria. The results indicate the potential of glass samples for bone tissue regeneration.

## Graphical abstract

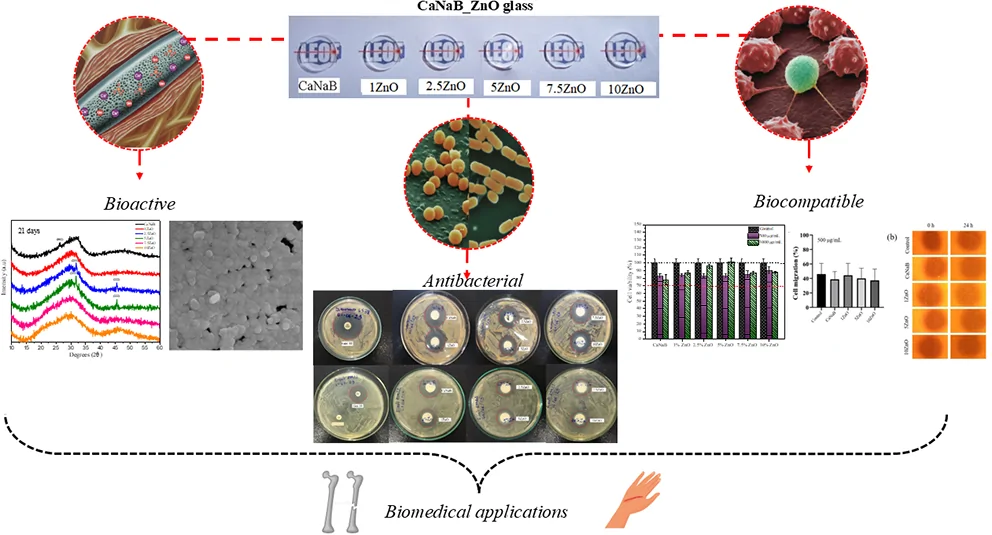

## 1 Introduction

Bioactive glasses are biomaterials developed and applied to repair and correct damaged tissues.^1–4^ These biomaterials may offer advantages over traditional methods for repairing bone and epithelial tissues, such as bioinert implants, autografts, xenografts, and allografts.^1,3–5^ These conventional methods have limitations such as disease transmission,^1^ donor site morbidity^3^ and limited availability.^1^

Bioactive glasses have been studied since 1969 when Larry Hench developed the first glass with bioactive properties, a silicate glass called Bioglass 45S5.^6–8^ The main properties of bioactive glasses are the ability to release therapeutic ions and form a hydroxyapatite layer on their surface when immersed in body fluids. This layer acts as a binding interface between the material and surrounding tissue.^9–11^ Silicate glasses form a SiO_2_-rich layer during *in vitro* or *in vivo* analysis. This layer decreases the ion release rate and slows the hydroxyapatite formation.^7,9,12^ Consequently, new glass compositions were explored to enhance bioactivity.^13–17^

Borate bioactive glasses have a faster and more complete degradation rate than silicate bioactive glasses when immersed in physiological fluids, due to their lower chemical durability and a higher ion release rate.^13,16^ Different elements can be added to the composition of borate glasses, which when inserted into the glass network, can modify and improve their physical, bioactive, and mechanical properties.^18^

For example, adding sodium oxide facilitates the melting and homogenization of glasses during synthesis^19^ and calcium oxide stimulates the formation of new bone tissue and promotes angiogenesis.^12,20^ Zinc is also a very promising element for this application, as it has antibacterial action,^9,18,21,22^ anti-inflammatory properties,^9,18^ stimulates homeostasis,^13^ angiogenesis,^13,22^ cell proliferation in the process of wound healing^13,23^ and also plays a role in osteogenesis and bone remodeling.^18,20,21,24^ Therefore, in this work, the role of ZnO incorporation in the bioactivity, structural changes, cytotoxicity, and antibacterial activity of the CaO-P_2_O_5_–Na_2_O–B_2_O_3_ glass system was evaluated.

## 2 Materials and Methods

### 2.1 Samples Preparation

Bioactive borate glasses with composition (64-*x*) B_2_O_3_–2P_2_O_5_–18CaO–16Na_2_O–xZnO, with *x* = 0, 1, 2.5, 5, 7.5, and 10 mol % were synthesized by the melt-quenching method. To synthesis the samples, B_2_O_3_, P_2_O_5_, CaCO_3_, Na_2_CO_3_, and ZnO (Sigma-Aldrich) with purity greater than 99% were used. The reagents were weighed on an analytical balance (AUW220D—Shimadzu), homogenized in an agate mortar, transferred to a platinum crucible, and melted in an electric furnace in air at 1150 °C. The melted material was poured into a stainless-steel mold previously heated at 450 °C. Subsequently, the vitreous samples were thermally treated for 4 h at 450 °C in a furnace with air atmosphere. Table 1 shows the identification codes and the compositions in molar percentage (mol %) of the samples synthesized.

**Table 1:** Sample Codes, Composition (%mol), Volumetric Density (ρ), and Molar Volume (*V*_m_) of the Samples Prepared

	B_2_O_3_	P_2_O_5_	CaO	Na_2_O	ZnO	ρ	*V*_m_
Sample code	%mol	%mol	%mol	%mol	%mol	g/cm^3^	cm^3^/mol
CaNaB	64	2	18	16	-	2.476	23.23
1ZnO	63	2	18	16	1	2.505	26.96
2.5ZnO	61.5	2	18	16	2.5	2.536	26.69
5ZnO	59	2	18	16	5	2.563	26.53
7.5ZnO	56.5	2	18	16	7.5	2.645	25.82
10ZnO	54	2	18	16	10	2.670	25.68

### 2.2 Characterizations

The volumetric density (ρ) and molar volume (*V*_m_) of the glass samples were obtained using the Archimedes principle described by Brauer et al.,^25^ using distilled water at 25 °C as immersion liquid. The differential thermal analysis (DTA) was carried out on a TG-DTA equipment (Shimadzu- model DTG-60) in nitrogen atmosphere. A mass of 20 mg of samples was placed into a platinum crucible in a temperature range from 20 to 900 °C, with a heating rate of 10 °C/min and a 100 mL/min flow of nitrogen. The values of glass transition temperature (*T*_g_), crystallization onset temperature (*T*_x_), and thermal stability (*T*_x_ – *T*_g_) of the glass samples were determined from the thermograms.

To the radiopacity study, digital radiographs of the samples were captured using the FOCUS X-ray device (KAVO), with radiation emission of 60 eV and 7 mA, focus-object distance of 20 cm, and exposure time of 0.160 s. An aluminum penetrometer (Figure 1 *)*, with 5 steps from 1 to 5 mm (mm) thick (1 mm each step), was radiographed beside the samples to compare the gray level. This comparison was carried out using the histogram tool in Adobe Photoshop Lightroom software. For this analysis, slices of the glass samples with a thickness of 1 mm were used.

**Figure 1:**
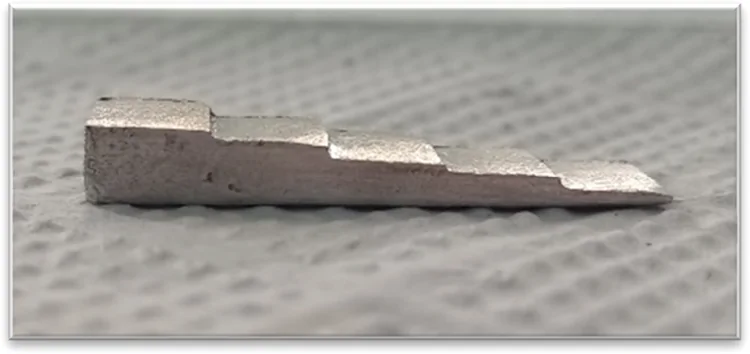
Aluminum penetrometer used in radiopacity analysis.

The evaluation of cytotoxicity of the glasses was carried out using RAW-264.7 murine macrophage line cells and using the 3-(4,5-dimethyl-2-thiazolyl)-2,5-diphenyl-2*H*-tetrazolium (MTT) assay.^26^ The tests were performed in triplicate. A one-way ANOVA (analysis of variance) with a significance level of 0.05 was used to check the significant difference between the means, and Tukey’s test was used to identify which means differ from each other.

The bioactivity tests were carried out using SBF, a simulated body fluid prepared according to the methodology proposed by Kokubo et al.^27^ The glass samples were cut into slices using a diamond saw cutter (*Nitrocut* brand) and grooves were made on their surface, rubbing them with 600 grit sandpaper using a polishing equipment (*Pantec* brand, *Panlipan U* model).

The average thickness of the slices was approximately 1.25 mm, with a diameter of 10 mm; the mass-to-surface area ratio for each sample was 1.238 × 10^–3^ g/mm^2^ for CaNaB, 1.229 × 10^–3^ g/mm^2^ for 1ZnO, 1.297 × 10^–3^ g/mm^2^ for 2.5ZnO, 1.254 × 10^–3^ g/mm^2^ for 5ZnO, 1.219 × 10^–3^ g/mm^2^ for 7.5ZnO, and 1.316 × 10^–3^ g/mm^2^ for 10ZnO. The *in vitro* tests have been performed according to the methodology described by Kokubo et al. During the immersion periods of 7, 14, 21, and 28 days, the SBF pH, in which the samples were soaked, was measured daily using a pHmeter (Gehaka -model PG 1800).

*In vitro* degradation was analyzed by the loss of mass presented by the glass samples during all periods of immersion in SBF.^28^ Mass loss was calculated using eq 1, where *w*_0_ is the mass of the sample before immersion in SBF and *w* is the mass after immersion.^29^

1 $$\Delta W=\frac{\left(W_{0}-W\right)}{W_{0}}.100$$

After 21 days of immersion in SBF, the samples were characterized morphologically by scanning electron microscopy (SEM) and chemically by energy-dispersive X-ray spectroscopy (EDS). The equipment used was a scanning electron microscope (movel Evo HD, ZEISS and detector SE, with 20000× image magnification) with an accoupled Bruker’s XFlash 410 M detector. From spectra, the calcium-to-phosphorus atomic ratio (Ca/P) ratio was calculated.

The X-ray diffraction (XRD) analysis was performed using an Empyrean equipment (Panalytical), with angular scanning from 10 to 60° (2θ), with a step of 0.02 in 2 s per step. The diffractograms were obtained before and after immersion of all samples in SBF.

Fourier transform infrared (FTIR) absorption spectra were obtained in a spectrometer (BRUKER—model Vertex 70 V) at room temperature, with an average of 32 scans and a spectral resolution of 4 cm^–1^, in a wavenumber range of 4000–400 cm^–1^, using the potassium bromide (KBr) pellet technique. FTIR spectra of the samples were also obtained after immersion in SBF. The fraction of tetracoordinated boron (*N*_4_) was calculated by deconvolution of the spectra for the samples before immersion in SBF and using the eq 2. *A*_4_ refers to the area of the bands corresponding to the BO_4_ groups and *A*_3_ refers to the area of the bands corresponding to the BO_3_ groups.^30^

2 $$N_{4}=\frac{A_{4}}{A_{4}+A_{3}}$$

The antibacterial activity of the glasses was evaluated using the agar disk diffusion methodology described by da Silva et al.^31^ in Gram-positive bacteria *Staphylococcus aureus* ATCC 6538 and Gram-negative bacteria *Escherichia coli* ATCC 25922. The tests were carried out in duplicate and the mean was calculated. In order to check whether there is a significant difference between the groups means, ANOVA- One way analysis of variance was used, at a significance level of 0.05; in order to identify which means differ from each other, the Tukey test was used.

The human fibroblast cell line CCD1072Sk was cultured in IMDM medium (Gibco), supplemented with 10% Fetal Bovine Serum (BRAND) and 1% Penicillin-Streptomycin (Gibco), in an incubator at 37 °C with 5% CO_2_. Subcultures were performed using 0.05% Trypsin-EDTA (Gibco) for cell detachment.

Bioactive glass extracts (CaNaB, 1ZnO, 2.5ZnO, 5ZnO, 7.5ZnO and 10ZnO) were prepared by incubating 3 mg of each material in 3 mL of IMDM medium for 24 h at 37 °C to create a stock solution of 1 mg/mL. After centrifugation (1300 rpm, 10 min), the supernatant was collected, constituting the 1000 μg/mL solution, which was subsequently diluted in IMDM to also obtain the 500 μg/mL concentration.

To evaluate the cytotoxicity of the extracts, CCD-1072Sk, human fibroblast cells, were seeded in 24-well plates and treated for 24 h with extracts of the different glasses at concentrations of 500 μg/mL. After 24, cell viability was assessed using the trypan blue exclusion method (Aldrich, 302643–25G), following the protocol by Crowley et al.^32^ The percentage of viable cells was determined by counting stained and unstained cells in a Neubauer chamber. The fibroblasts were kindly provided by Professor Mauricio Baptista da Silva, from the Laboratory of Photoinduced Processes and Interfaces, Institute of Chemistry (IQ), University of São Paulo (USP). Cells were confirmed to be mycoplasma-free, and were originally obtained from the Cell Bank of Rio de Janeiro (BCRJ, Brazil) under the catalog number 0062.

Cell Migration Assay (Scratch Assay) were analyzed using the fibroblasts which were seeded in 24-well plates (2 × 10^4^ cells/mL) and cultured for 48 h. After the first 24 h, a scratch was created in each monolayer using a 200 μL pipet tip. The cells were then treated with the glass extracts (500 and 1000 μg/mL) or IMDM medium as a control. Gap closure was photographed at 0, 6, and 24 h after its creation using an inverted microscope equipped with a digital camera (sCMEX-5, EUROMEX). The gap area was quantified using ImageJ software, and data were statistically analyzed with GraphPad Prism (Version 8.0.2 (263)).

## 3 Results and Discussion

Figure 2 shows the disc-shaped samples measuring of 10 mm in diameter and 1.25 mm in thickness. All samples are transparent, colorless, and free of visible crystallites. The incorporation of ZnO did not produce any visible changes in the appearance of the glasses.

**Figure 2:**
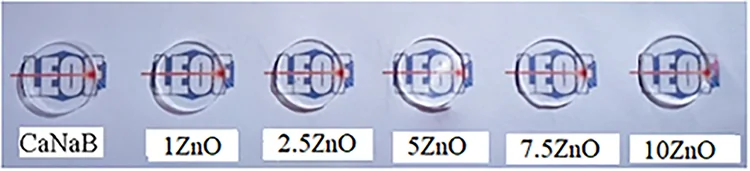
Glass samples with 10 mm in diameter and 1.25 in thickness.

### 3.1 Volumetric Density and Molar Volume

Figure 3 shows the volumetric density and molar volume values, as a function of the ZnO concentration in mol %, for the glass samples. The results show a linear increase in density with the ZnO content. Volumetric density (presented in Table 1) increased 7.66% from the CaNaB to the 10ZnO, this increase can be attributed to the replacement of B_2_O_3_ by ZnO in the composition of the samples, as the molar mass of ZnO (81.38 g/mol) is greater than that of boron trioxide (69.62 g/mol). This increase may also be associated with glass network contraction, a behavior indicated by the decrease in molar volume due to the increase in zinc oxide concentration.^33^

**Figure 3:**
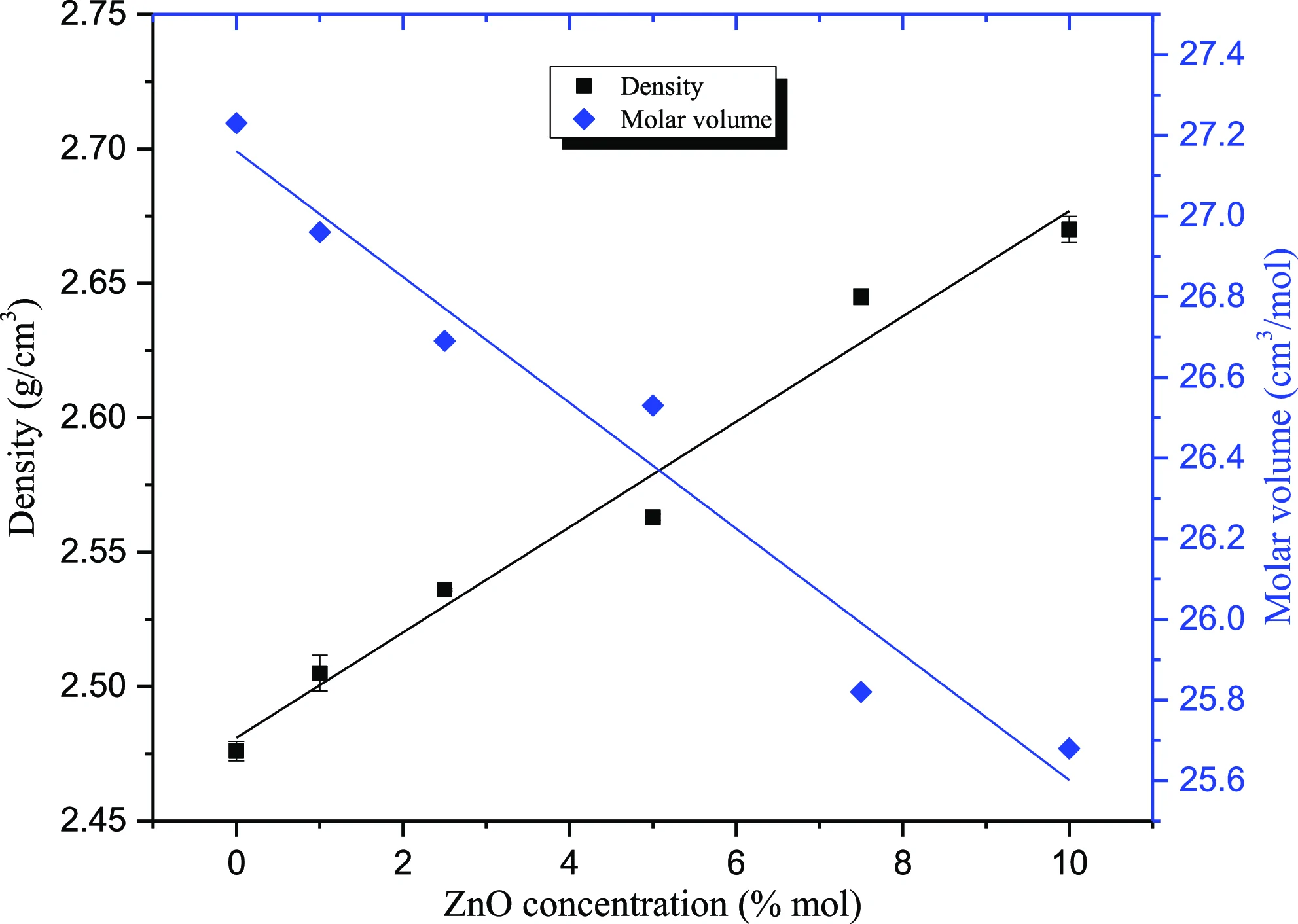
Density and molar volume of samples as a function of ZnO concentration.

The molar volume values show the inverse behavior to the density results; a decrease of 5.69% in the molar volume of the samples was observed with ZnO concentration increase. This behavior indicates that the insertion of ZnO made the glass network more contracted with a reduction in the bond size between the atoms.^34^

The molar volume is related to the spatial distribution of atoms in the glass network.^35^ Furthermore, the decrease in molar volume may have occurred due to a glass network compaction caused by the reduction of nonbridging oxygens (NBO).^30,35^ Ahmad et al. obtained a similar result for molar volume in a study with borate glasses replacing B_2_O_3_ by ZnO in different concentrations.^34^

### 3.2 X-ray Diffraction

Figure 4 shows the XRD patterns of the prepared glass samples. The diffractograms confirm the amorphous nature of the material, as indicated by the absence of crystalline peaks and the presence of only two broad amorphous halos, centered at ∼29 and 47° 2θ, which are characteristic of borate glasses.^15,36^ The XRD patterns of all samples exhibit similar features, suggesting that ZnO incorporation did not cause detectable structural changes within the resolution limits of X-ray diffraction.

**Figure 4:**
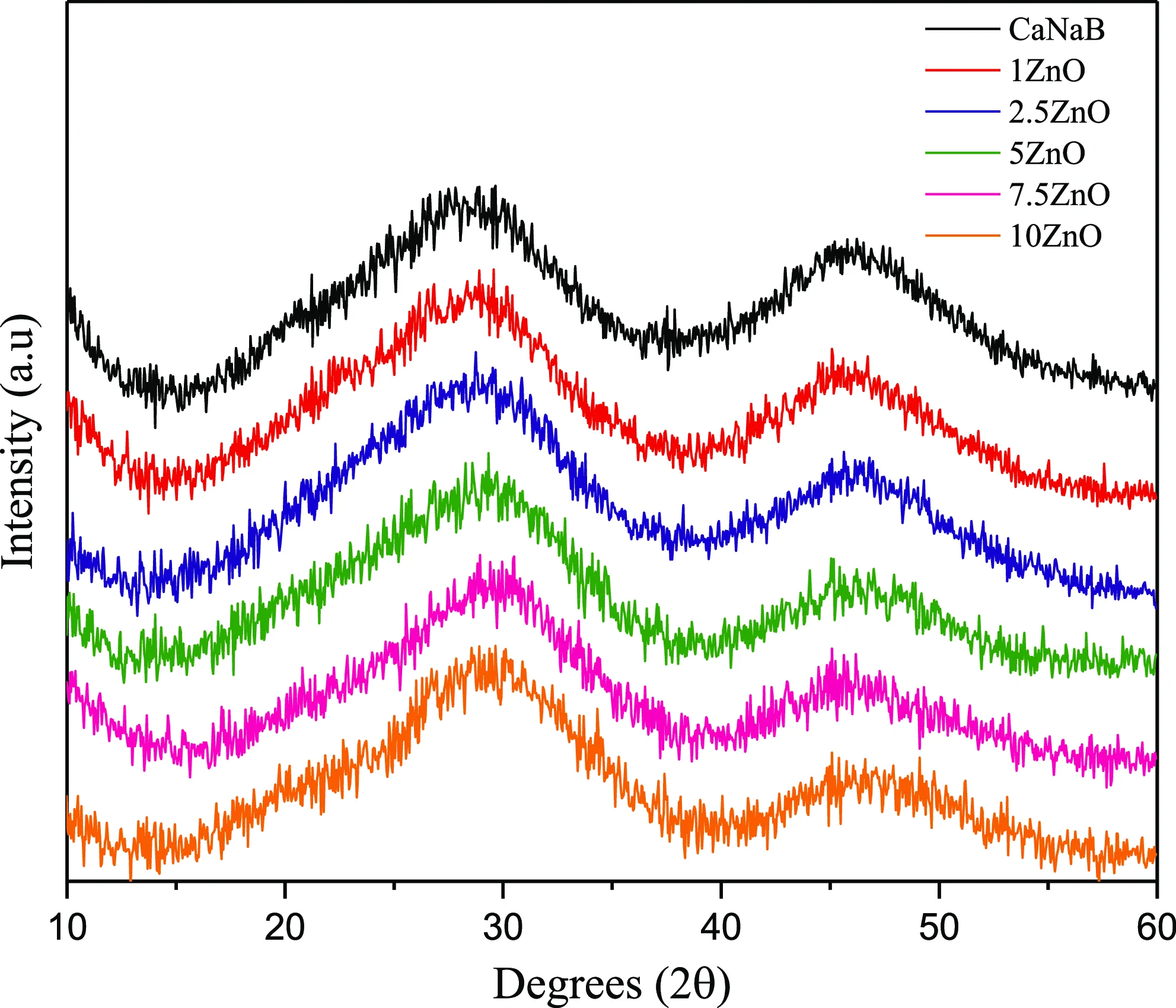
Diffractograms obtained for CaNaB, 1ZnO, 2.5ZnO, 5ZnO, 7.5ZnO, and 10ZnO samples.

### 3.3 Fourier Transform Infrared Spectroscopy

The FTIR absorption spectra of the glass samples are shown in Figure 5. Three main absorption regions can be distinguished, centered approximately within the ranges: the first region at ∼470–780 cm^–1^, the second at ∼780–1200 cm^–1^, and the third at approximately 1200–1650 cm^–1^.

**Figure 5:**
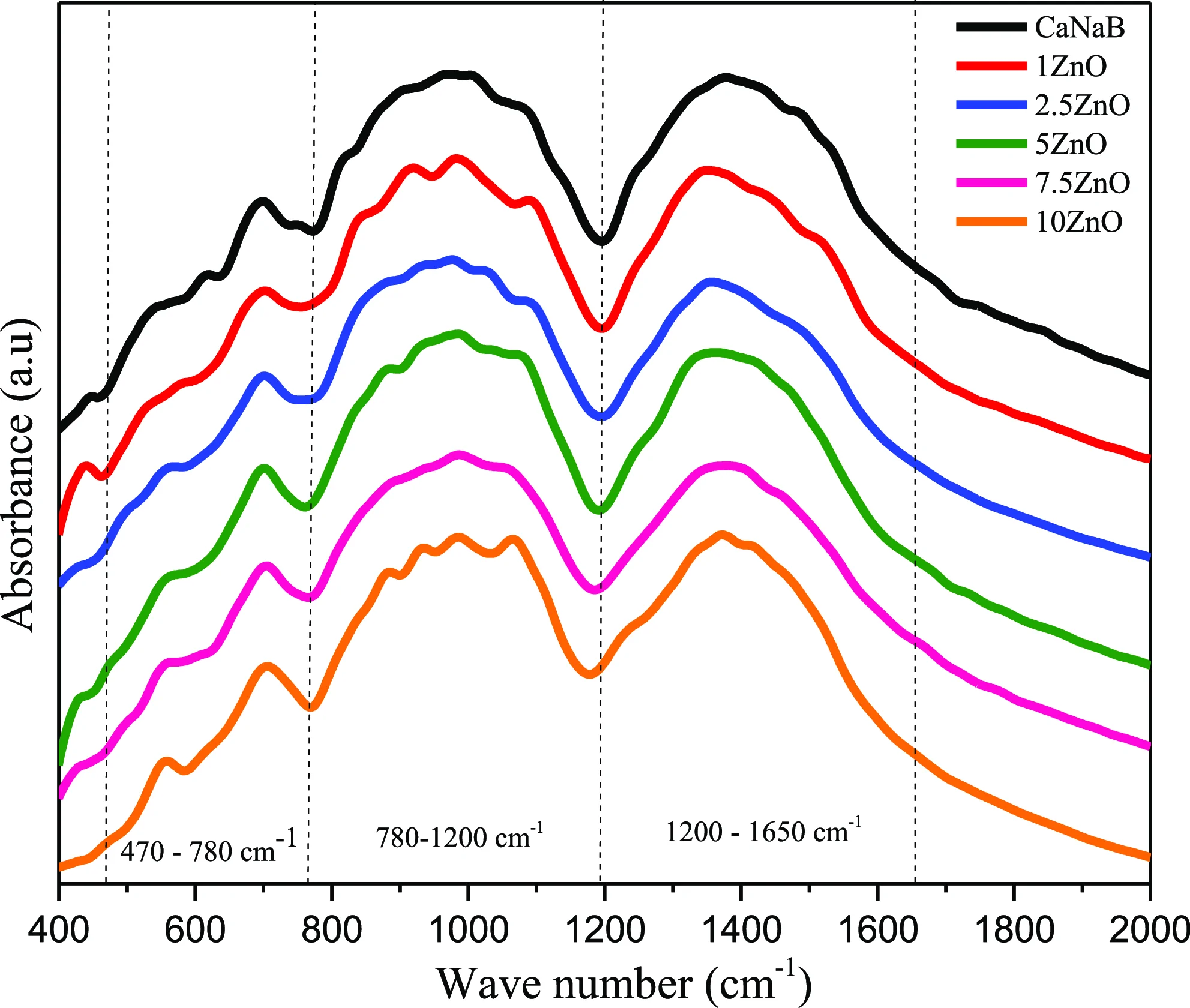
FTIR spectra of the CaNaB, 1ZnO, 2.5ZnO, 5ZnO, 7.5ZnO, and 10ZnO samples.

In the first region of the spectra, the band centered at approximately 560 cm^–1^ is attributed to P–O–P bond vibrations in the PO_4_^3–^ groups.^37,38^ The band in the region from 600 to 780 cm^–1^, with a center at ∼700 cm^–1^, is related to bending vibrations in the B–O–B bond in BO_3_ groups.^11,39^ The band in the central section of the spectra, from 780 to 1200 cm^–1^, is characteristic of stretching vibrations of the B–O–B bonds in BO_4_ groups.^40,41^ In the last region of spectra, the band at 1200–1650 cm^–1^ refers to stretching vibrations of the B–O–B bonds in BO_3_ groups.^37–39^

The deconvolution of FTIR spectra using Gaussian functions was performed to obtain the fraction *N*_4_ for the samples (Figure 6b) shows the *N*_4_ values, calculated for each sample, depending on the concentration of ZnO in the vitreous materials.

**Figure 6:**
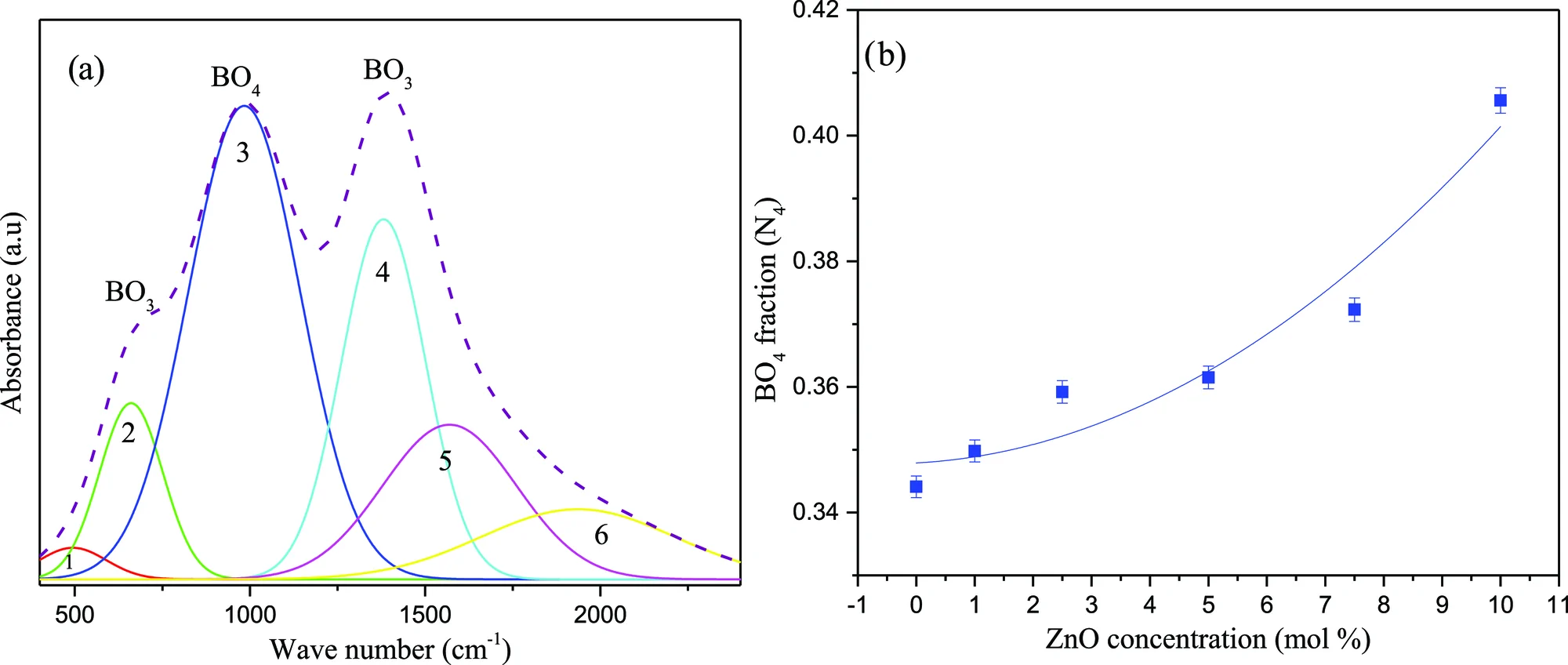
(a) Deconvolution of the FTIR spectrum of the 10ZnO sample; (b) N_4_ values as a function of the ZnO concentration in the glass samples.

In Figure 6b, an increase in the fraction of tetracoordinated borons (*N*_4_) can be observed as a function of ZnO concentration. This result indicates that the ZnO incorporation into the glass matrix provided a structural rearrangement leading to the conversion from BO_3_ to BO_4_ units, as suggested by the molar volume result. Therefore, the conversion of BO_3_ to BO_4_ units resulted in a smaller amount of the nonbridging oxygens present in the glass matrix^34,42^ and in the contraction of the glass network due to the addition of ZnO.^30^

### 3.4 Cellular Viability Analysis

Figure 7 shows the percentage of cell viability of RAW 247 murine macrophages after contact for 24 h with solution resulting from the dissolution of each vitreous sample in RPMI medium at concentrations of 500 μg/mL and 1000 μg/mL. According to ISO 10993, for a material to be considered noncytotoxic, a minimum cell viability of 70% is allowed.^43^ Therefore, all glass samples are noncytotoxic at the two concentrations tested, as they presented cell viability greater than 70%.

**Figure 7:**
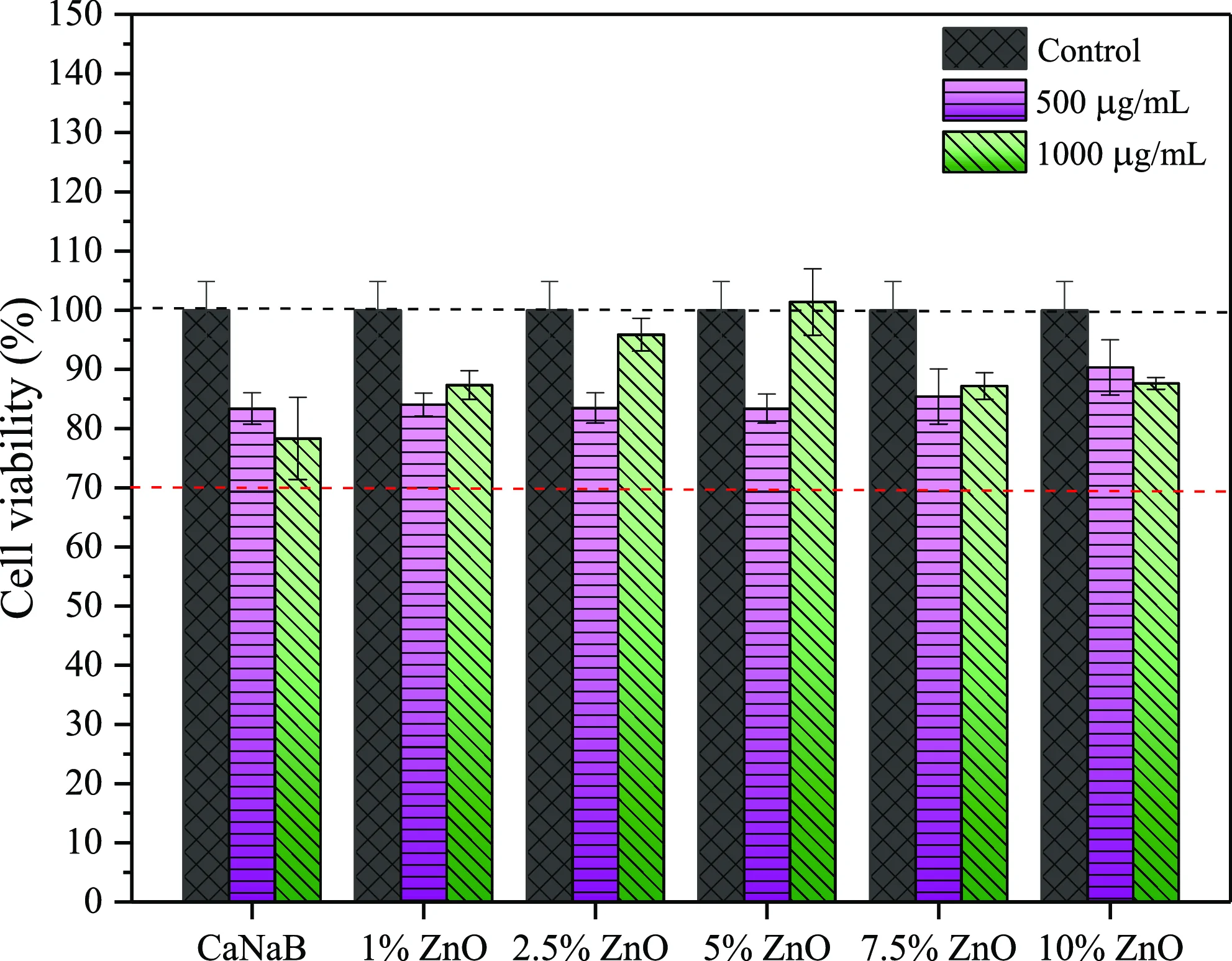
Cellular viability of RAW 247 murine macrophages after contact for 24 h with vitreous extracts of samples at concentrations of 500 μg/mL and 1000 μg/mL.

It can also be observed that the percentages of cell viability obtained after exposure of macrophages to glass extracts, at a concentration of 500 μg/mL, were similar, without significant differences with the rise in the concentration of ZnO added to the material. At a concentration of 1000 μg/mL, there was an increase in cell viability with the addition of ZnO up to 5% in mol in the vitreous matrix, followed by a decrease in viability for the samples 7.5ZnO and 10ZnO.

However, considering the ANOVA test (with a significance level of 0.05, a 95% confidence interval, an *F*-value of 1.8405, and a *p*-value of 0.1792), there is no significant difference between the cell viability values obtained for all samples at concentrations of 500 μg/mL and 1000 μg/mL.

### 3.5 Differential Thermal Analysis

The values of *T*_g_, *T*_x_, and thermal stability (*T*_x_ – *T*_g_) obtained for the samples are shown in Table 2 and Figure 8.

**Figure 8:**
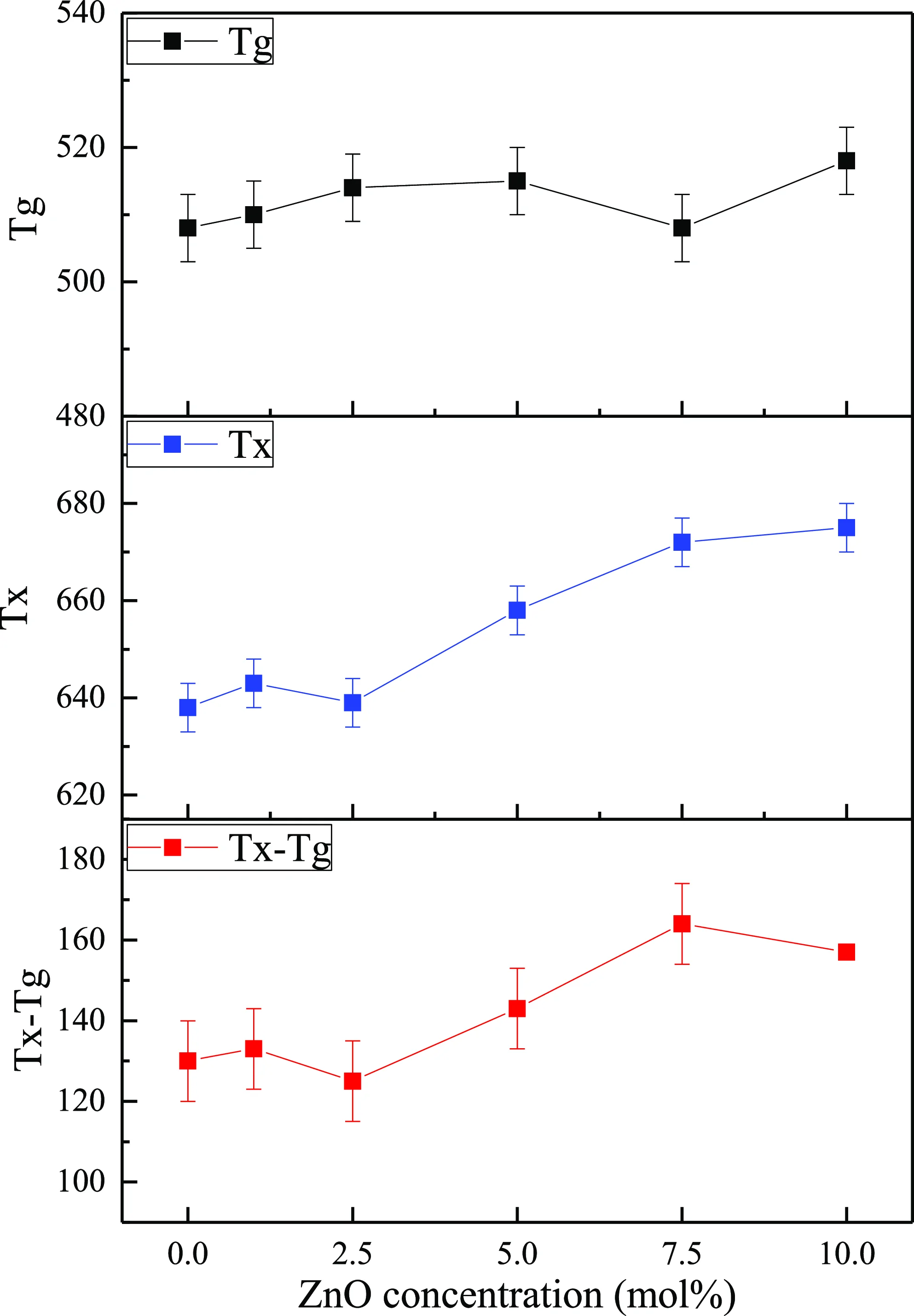
Values of *T*_g_, *T*_x_, and *T*_x_ – *T*_g_ as a function of the ZnO concentration.

**Table 2:** Values of *T*_g_, *T*_x_, and *T*_x_ – *T*_g_ of the Glass Samples Synthesized

Sample	*T*_g_ (°C) (±5 °C)	*T*_x_ (°C) (±5 °C)	*T*_x_ – *T*_g_ (°C) (±10 °C)
CaNaB	508	638	130
1ZnO	510	643	133
2.5ZnO	514	639	125
5ZnO	515	658	143
7.5ZnO	508	672	164
10ZnO	518	675	157

From Table 2 and Figure 8, it is possible to indicate that the *T*_g_ of the vitreous samples does not vary significantly within the errors. The *T*_x_ values for the 5ZnO, 7.5ZnO, and 10ZnO samples increased compared to the CaNaB sample. Thermal stability shows a similar behavior, increasing for the 5ZnO, 7.5ZnO, and 10ZnO samples.

The greater the range of glass thermal stability, the lower the probability of crystallization during thermal processing.^44^ Therefore, the increase in thermal stability with increasing ZnO content broadens the working temperature range, which is advantageous for applications in biomedical devices, such as the coating of titanium implants.

Kullberg et al. reported results similar to those obtained in the present study, observing that both *T*_x_ and the thermal stability of ZnO-containing borosilicate glasses increased with increasing ZnO/B_2_O_3_ ratio.^44^

Naresh et al. also observed that the addition of ZnO at concentrations of 0.5, 10, 15, and 20 mol % increased the glass network rigidity and thermal stability of the boro-tellurite glasses they studied.^45^

### 3.6 Radiopacity Analysis

From the radiography taken (Figure 9a), the gray level values were obtained for each of the steps of the penetrometer and the glass samples. The steps have a thickness of 1 mm (A), 2 mm (B), 3 mm (C), 4 mm (4) and 5 mm (E). By comparing the gray level of the glass samples with the gray level of the aluminum steps, it was possible to obtain the radiopacity of the CaNaB, 1ZnO, 2.5ZnO, 5ZnO, 7.5ZnO, and 10ZnO samples in millimeters of aluminum equivalent (mmAl) (shown in Figure 9b).

**Figure 9:**
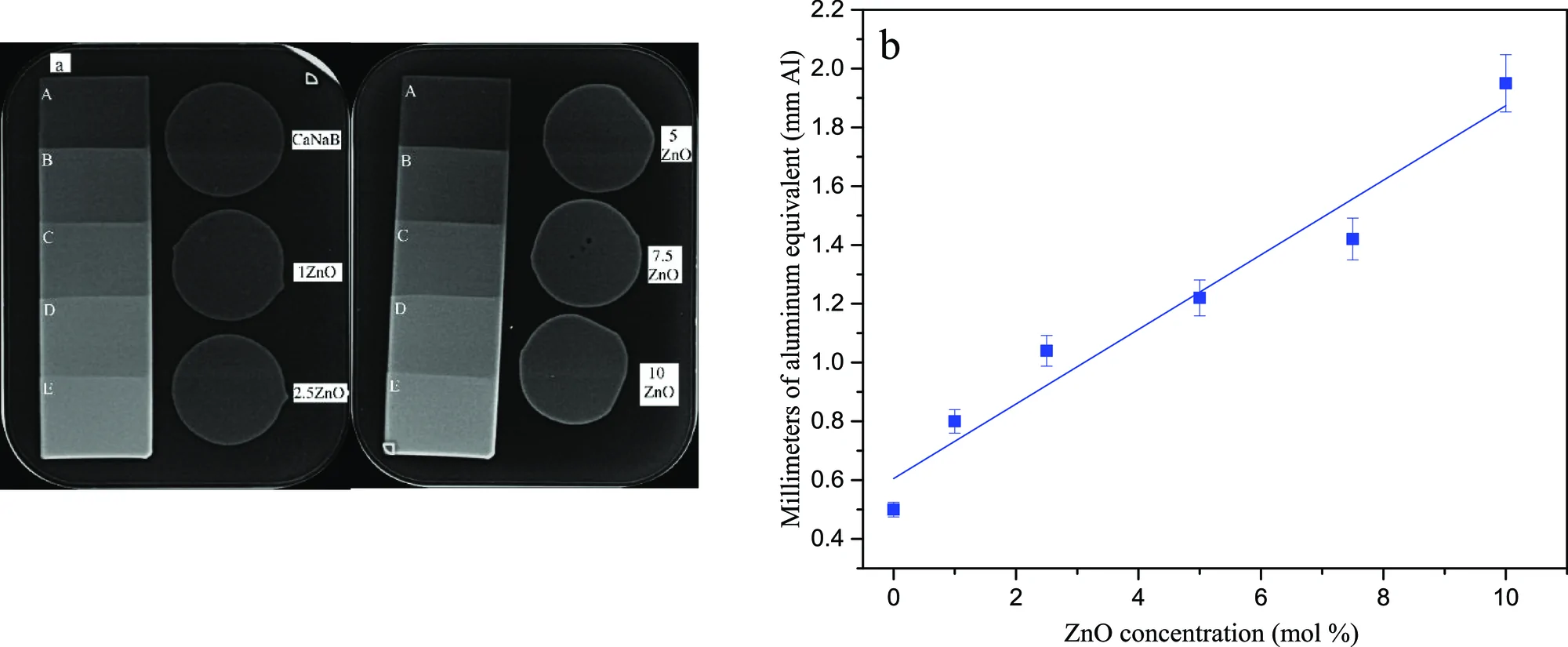
(a) Radiograph of the glass samples and aluminum penetrometer; (b) radiopacity level of samples, in mmAl, as a function of ZnO concentration.

In Figure 9b, it is possible to observe that the CaNaB sample presented a radiopacity level equivalent to 0.5 mmAl, the sample 1ZnO is equivalent to 0.8 mmAl, the 2.5ZnO is equivalent to 1.04 mmAl, 5ZnO is equivalent to 1.22 mmAl, 7.5ZnO is equivalent to 1.42 mmAl and 10ZnO is equivalent to 1.95 mmAl. Therefore, it is possible to indicate an increase in the radiopacity of the glasses due to the increase in the concentration of ZnO in the vitreous matrix. This increase occurs because the zinc atomic number is higher than the boron atomic number.^46^

In radiographic images used to monitor the process of material implantation in bone and dental tissues, it is necessary to distinguish the material from the surrounding tissue. According to ISO 6876:2001, root canal sealing materials require a minimum radiopacity of 3 mmAl/eq This requirement would preclude the use of the glasses from this study for that purpose.^47^ However, dentin exhibits a radiopacity equivalent to 1 mmAl;^48^ with the incorporation of higher ZnO concentrations, radiopacity values exceeding 1 mmAl were observed for the 5ZnO, 7.5ZnO, and 10ZnO samples. This demonstrates that, in dental applications, these bioactive borate glasses could likely be distinguished from dentin when monitored via radiography.

### 3.7 pH Values Analysis

The pH values of the SBF solution, containing the glass samples CaNaB, 1ZnO, 2.5ZnO, 5ZnO, 7.5ZnO, and 10ZnO, are shown in Figure 10. The variations in pH values indicated an ion exchange between the glasses and the SBF over the periods of 7, 14, 21, and 28 days of immersion.

**Figure 10:**
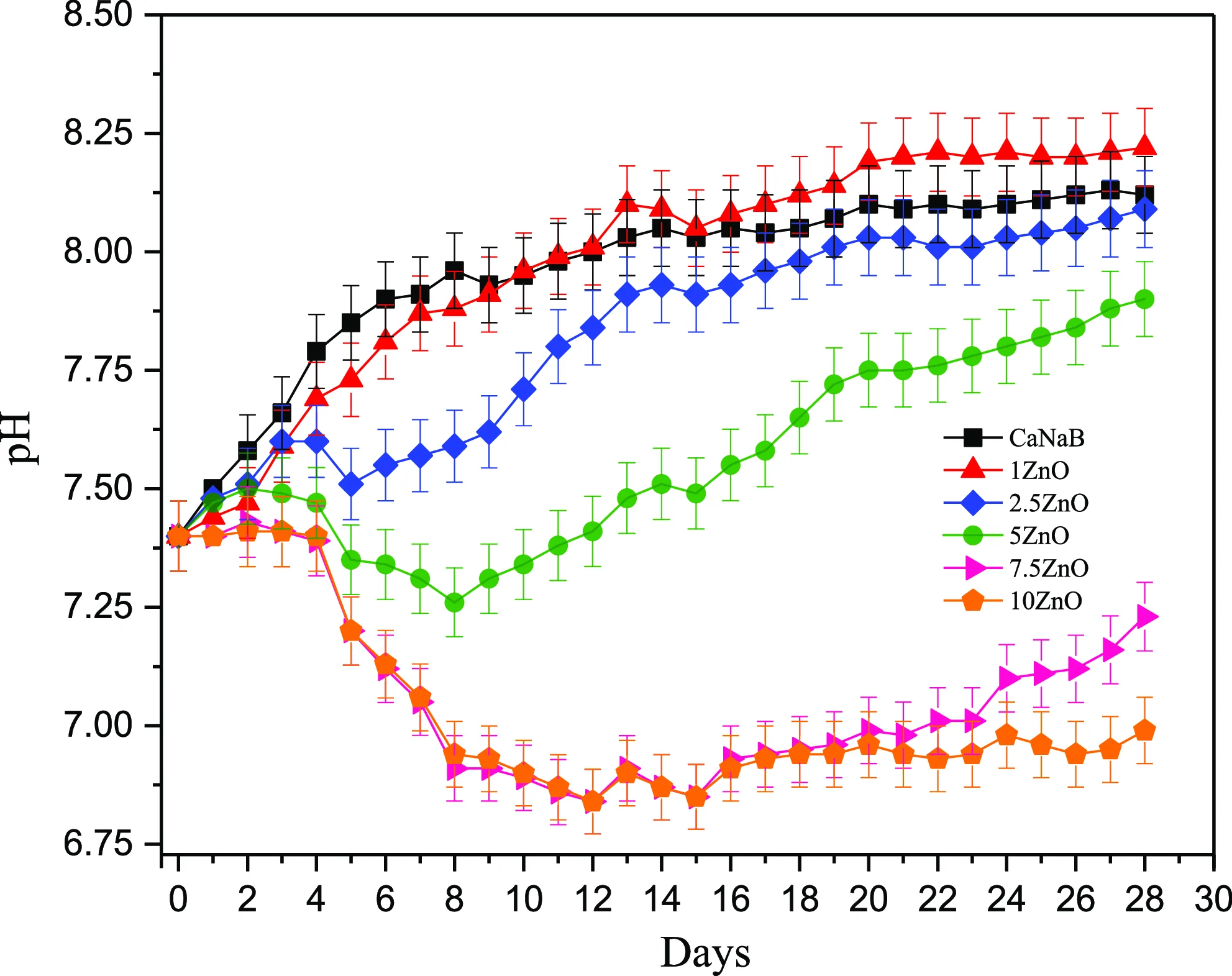
SBF pH values measured during the 28 days of immersion for the CaNaB, 1ZnO, 2.5ZnO, 5ZnO, 7.5ZnO, and 10ZnO samples.

It is possible to observe that, over the days, the pH values of the SBF showed different behavior according to the immersed sample. The pH of the SBF of the CaNaB and 1ZnO samples increased over the days until 20th day, followed by stabilization. This increase was associated with the ionic exchange between Ca^2+^ and Na^+^ ions from the glass and the H^+^ ions from the SBF solution. This ionic exchange increases the concentration of alkaline ions and decreases the concentration of H^+^ ions in the immersion environment.^49–51^

The pH of the SBF in which the 2.5ZnO sample was immersed remained nearly stable until the ninth day of immersion. From the 10th to the 13th day, the pH values gradually rose. After 13th, the pH values showed a stabilization tendency. For the 5ZnO sample, a reduction in SBF pH was observed until the eighth day, followed by a subsequent increase until the 28th day. The pH of the solutions containing the samples 7.5ZnO and 10ZnO showed similar behavior. These showed a more pronounced decrease until the 12th day and subsequently, the 7.5ZnO sample showed a subtle increase in pH, while the 10ZnO sample maintained a constant pH until the 28th day.

The decrease in pH values can be associated with the increase in the concentration of ZnO in the composition; it is possible that there was a greater migration of zinc ions to the SBF and this favored the formation of Zn­(OH)_2_ in the solution, increasing the concentration of free H^+^ in the medium, causing a decrease in the SBF’s pH.^52–54^ The formation of Zn­(OH)_2_ in the solution^55^ can lead to a decrease in the rate of hydroxyapatite formation due to the competition of Zn^2+^ ions with calcium phosphate for OH^–^ ions; ions that are necessary in the final crystallization phase of HA.^7,56^

It can also be inferred that, due to the increased stability of the material with the addition of ZnO, there was less exchange of Ca^2+^ and Na^+^ ions from the glass with H^+^ ions from the solution, as evidenced by the observation of lower pH values.^49,50^ As these ion exchanges are important for hydroxyapatite formation in the material, a reduction in the ion-exchange rate can lead to a decrease in the HA layer formation rate.^7^

### 3.8 *In Vitro* Degradation Analysis

Figure 11 shows the percentages of mass loss of all samples over 28 days of immersion. It is possible to observe that the percentage of mass loss tends to increase as the immersion time progressed for the CaNaB, 1ZnO, and 2.5ZnO samples until the 28th day of immersion and for the 5ZnO, 7.5ZnO, and 10ZnO until the 21st day. It is also noticed that, starting from the 2.5ZnO sample, with increasing ZnO content, there is a decrease in the percentage of mass loss.

**Figure 11:**
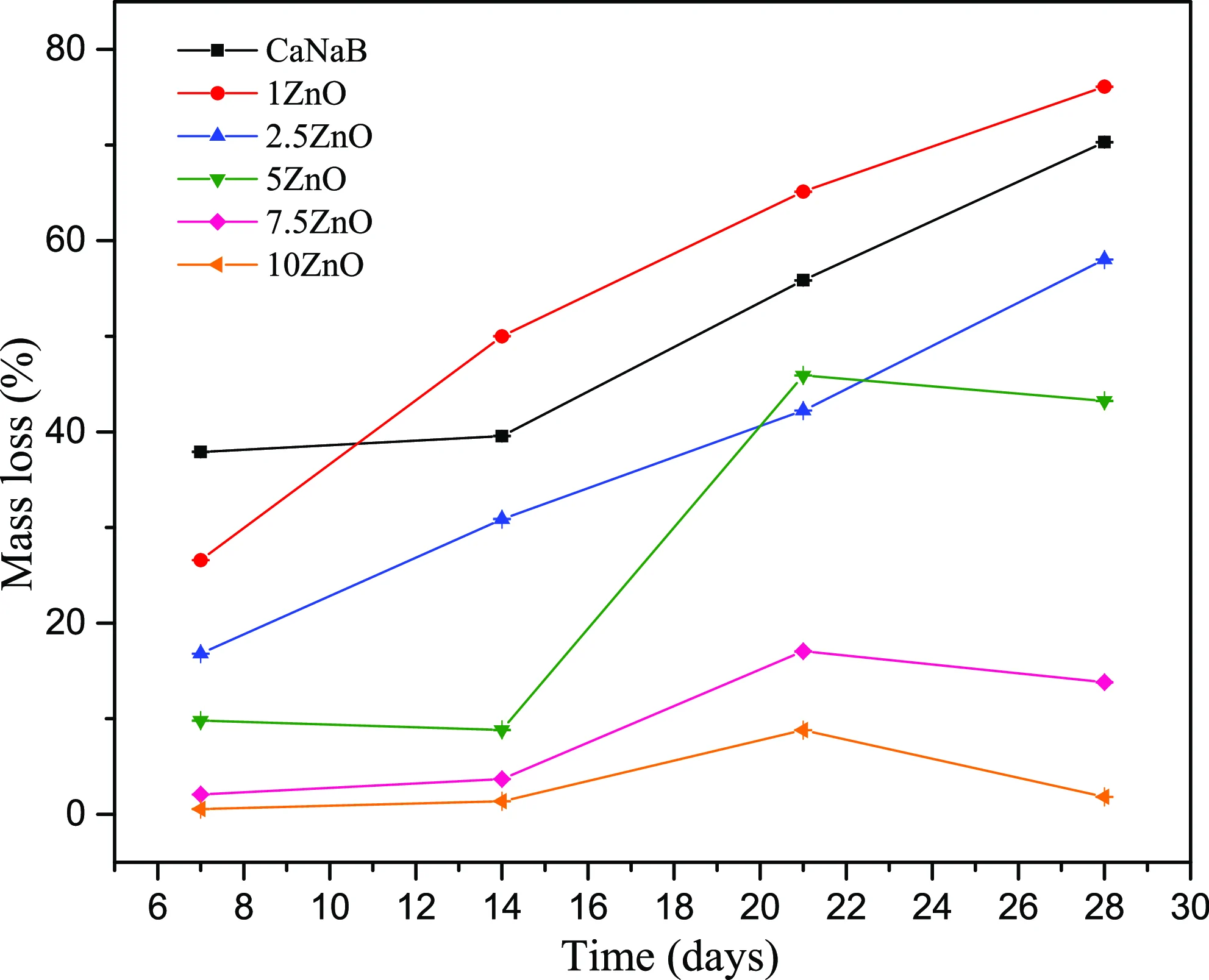
Mass loss percentage of samples as a function of immersion time.

The incorporation of higher ZnO concentrations into the glass matrix can increase the chemical stability of the material, as supported by the molar volume and BO_4_ fraction (*N*_4_) results. This causes a reduction in the dissolution rate of glasses due to the decrease in the leaching of ions from the samples into the medium, consequently delaying their degradation.^57^

In addition, mass loss percentage results (Figure 11) and the pH measurements of the SBF containing the samples (Figure 10) are directly related. Both pH and mass loss percentages decreased with increasing ZnO content. These findings indicate that the chemical and physical degradation processes of the samples are directly related to the ionic exchanges between the material and the SBF solution.

### 3.9 XRD after Immersion in SBF

Figure 12 shows the diffractograms for CaNaB and 5ZnO samples after 7, 14, 21, and 28 days of immersion in SBF. In the diffractograms for the CaNaB sample (Figure 12a), it was possible to observe the appearance of peaks at 26 and 32° 2θ after all immersion periods, as well as a peak at 53° 2θ in the diffractograms obtained after 7 and 28 days of immersion in SBF. The peaks at 26, 32 and 53° 2θ correspond to planes (002), (211) and (004) of the hydroxyapatite standard diffractogram (JCPDS 09–0432).^58–62^

**Figure 12:**
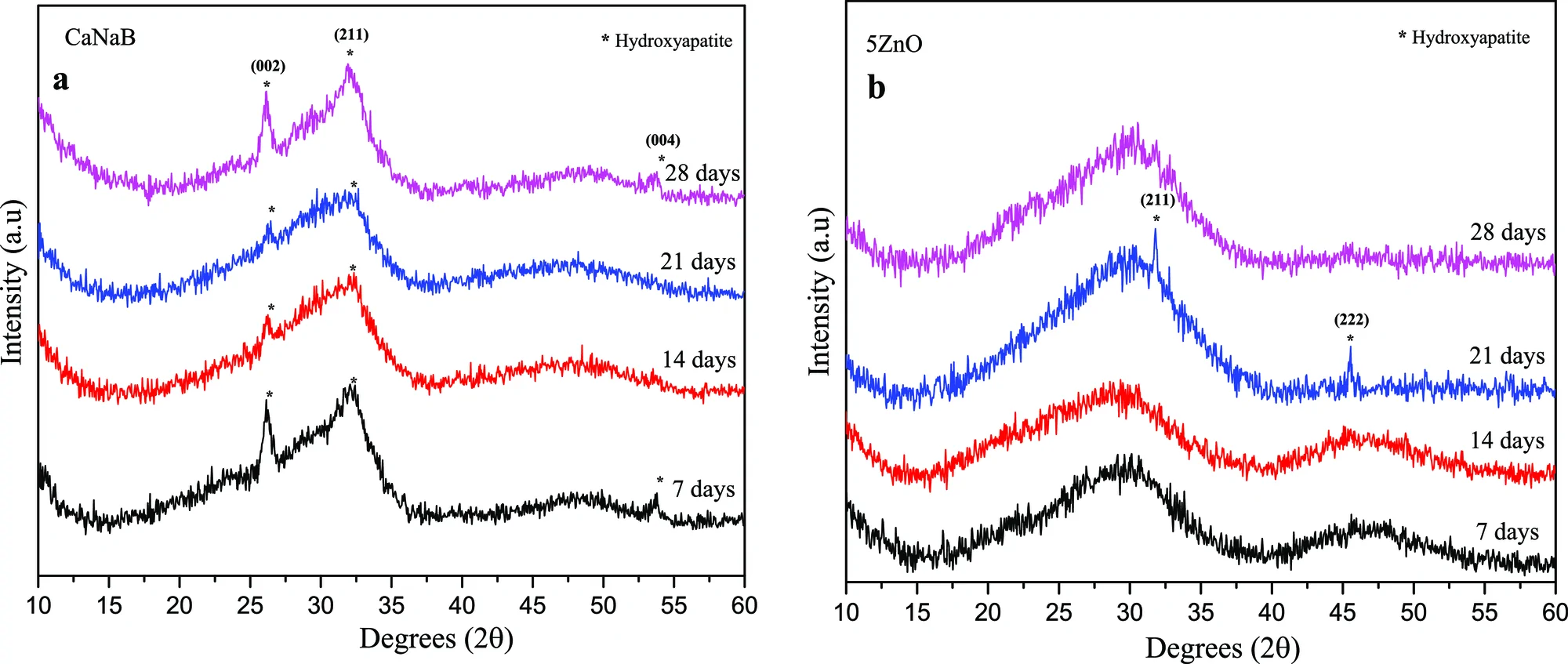
Diffractograms after 7, 14, 21, and 28 days of immersion in SBF for samples (a) CaNaB and (b) 5ZnO.

For the 5ZnO sample (Figure 12b), the appearance of crystalline peaks at 32 and 46° 2θ was observed only in the diffractogram for the sample immersed for 21 days in SBF. The peak at 46° corresponds to the plane (222) of the hydroxyapatite standard diffractogram.^61^ It was not possible to observe peaks in the diffractograms after the other immersion periods. The 2.5ZnO sample showed similar diffractograms to those of 5ZnO.

Figure 13 shows the diffractograms of all samples after 21 days of immersion. It is observed that only the CaNaB, 2.5ZnO, and 5ZnO samples presented crystalline peaks. The diffractograms of the 1ZnO, 7.5ZnO, and 10ZnO samples did not show peaks after all immersion periods.

**Figure 13:**
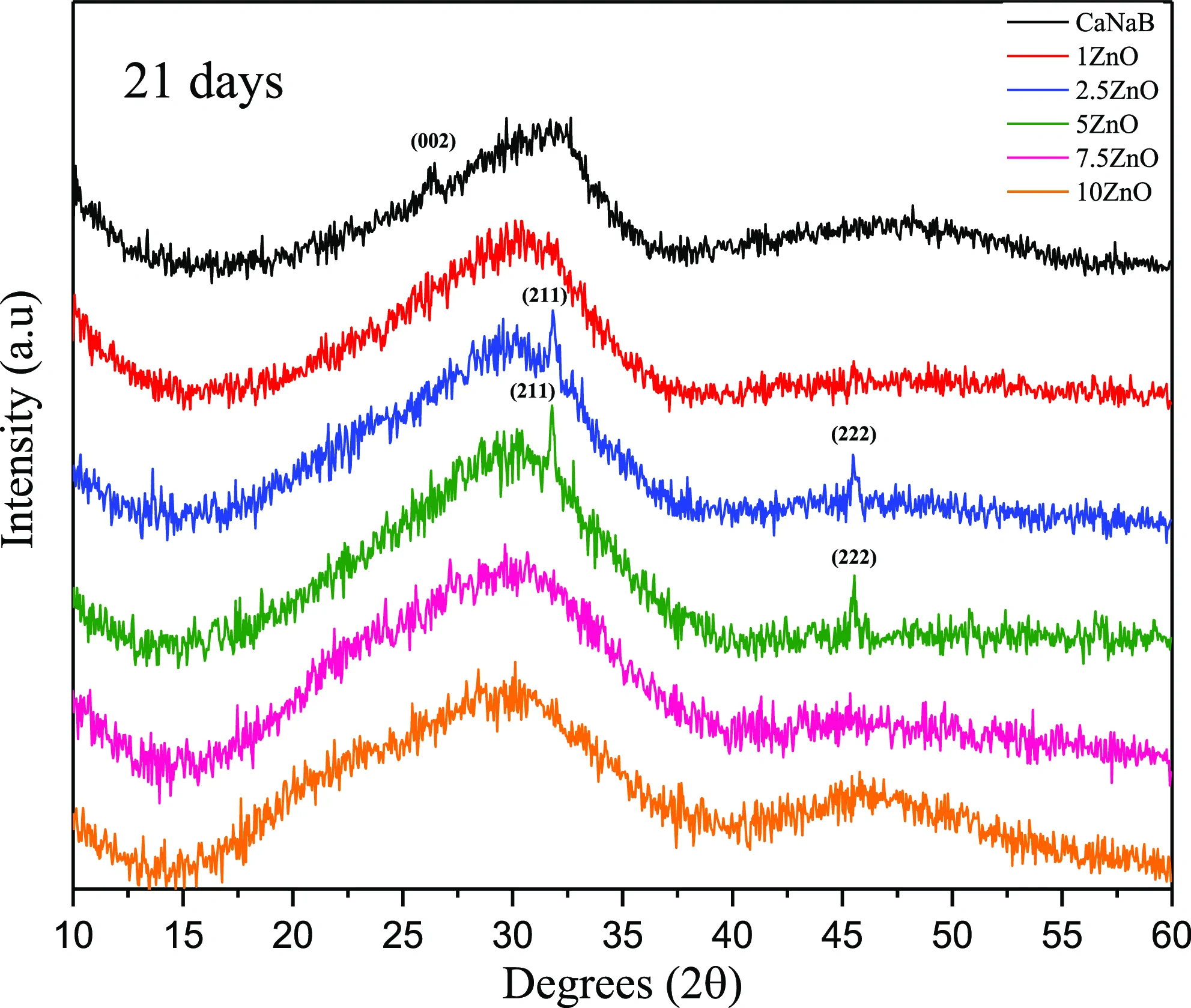
Diffractograms of CaNaB, 1ZnO, 2.5ZnO, 5ZnO, 7.5ZnO, and 10ZnO samples after immersion in SBF for 21 days.

The observed peaks coincide with the planes of the hydroxyapatite standard diffractogram. Thus, these data indicate that a crystalline hydroxyapatite layer was formed on the surface of the CaNaB, 2.5ZnO, and 5ZnO samples. They are indicating the bioactivity of the material.

The formation of hydroxyapatite on the glass surface occurs due to several dissolution and precipitation reactions. In this process, the Ca^2+^ ions that are on the sample surface, due to its dissolution process, react with the PO_4_^3–^ ions of the SBF, causing the formation of a calcium phosphate surface layer that can crystallize into HA through the incorporation of OH^–^ ions.^7,56^

If, after the formation of the HA layer, the solution still contains a large amount of H_3_O^+^ and OH^–^ ions, new precipitation reactions will occur, forming a new layer of amorphous calcium phosphate.^14^ This may have been the cause of the absence of peaks characteristic of hydroxyapatite in the diffractograms of the 1ZnO after all immersion periods, and in the diffractograms of the 2.5ZnO and 5ZnO samples after an immersion period of 28 days, even though these were observed after 21 days of immersion.

In the diffractograms of the 7.5ZnO and 10ZnO samples, crystalline peaks were not observed due to the greater stability of the glass network. An increase in the stability of the glass network was observed with the addition of ZnO, as indicated by density and molar volume results, as well as by the N_4_ calculation. This enhancement in chemical stability stemmed from the contraction of the glass network, driven by the transformation of BO_3_ units into BO_4_ units and a reduction in nonbridging oxygens within the network. A more compact glass network with a lower content of nonbridging oxygens exhibits greater chemical durability and a slower ion-exchange rate, consequently resulting in slower formation of HA.

### 3.10 FTIR Analysis after Immersion in SBF

Figure 14 shows the FTIR spectra of the CaNaB, 1ZnO, 2.5ZnO, 5ZnO, 7.5ZnO, and 10ZnO samples after 21 days of immersion in SBF.

**Figure 14:**
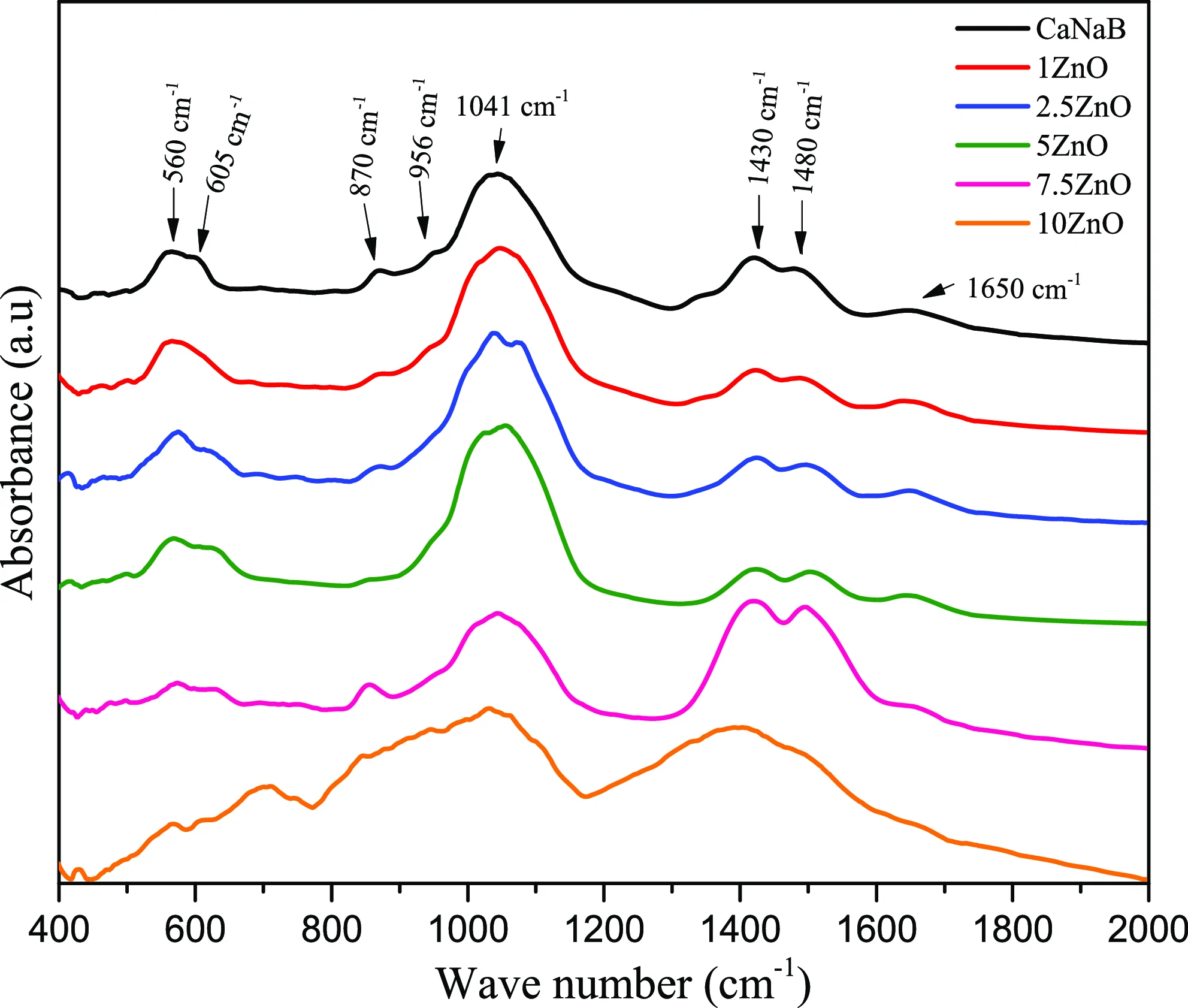
FTIR spectra of CaNaB, 1ZnO, 2.5ZnO, 5ZnO, 7.5ZnO, and 10ZnO samples after immersion in SBF for 21 days.

The band in the region of 500–650 cm^–1^, which in some spectra appears in the form of two bands with centers at 560 and 605 cm^–1^, is attributed to triply degenerate modes of bending vibrations of the O–P–O bonds in tetrahedral phosphate groups (PO_4_). This band corresponds to calcium phosphate and its division into two smaller bands with centers at 560 and 605 cm^–1^ is indicative of the crystallization of calcium phosphate.^63–66^

The most intense band centered at 1041 cm^–1^ is attributed to triply degenerate modes of the asymmetric stretching vibrations of O–P–O bonds in phosphate groups (PO_4_).^37,40,65^ The bands at 870 and 956 cm^–1^ are also due to the PO_4_ group. The bands at approximately 1430 and 1480 cm^–1^ are attributed to the resonance of the C–O bond in CO_3_^2–^ groups^64,67,68^ and the band with a center at approximately 1650 cm^–1^ refers to the OH^–^ groups.^53,59^ The presence of the bands at 500–650 cm^–1^ and 1041 cm^–1^ is indicative of the formation of the hydroxyapatite layer on the surface of the samples, confirming the bioactivity of the material.

Notably, with the increase in the concentration of ZnO, there is less definition and intensity of the characteristic PO_4_ bands and the bands referring to the borate groups are broader, especially in the spectra of the 10ZnO sample. These results indicate that higher concentrations of ZnO added to the glass samples can decrease the rate of HA formation. This reduction in the rate of HA formation may be related to the decrease in the dissolution of the glasses due to the increase in the chemical stability as a consequence of the greater network connectivity due to the insertion of zinc in the glass matrix.^9^

Neščáková et al. studied the influence of the addition of 5% ZnO (mol %) in mesoporous glasses. They observed that the glass containing zinc oxide did not present characteristic bands of the formation of hydroxyapatite (560 and 603 cm^–1^) after immersion in SBF for 14 days, different than was observed for glasses with no zinc.^66^

### 3.11 SEM-EDS Analysis

SEM and EDS analyses were performed to evaluate the surface of the samples after immersion in SBF solution for 21 days. The Figure 15 shows the SEM images and EDS spectra of the glass samples: (A) CaNaB, (B) 1ZnO, (C) 2.5ZnO, (D) ZnO, (E) 7ZnO, and (F) 10ZnO. The data show that all samples exhibit deposition of particles with a spherical morphology.

**Figure 15:**
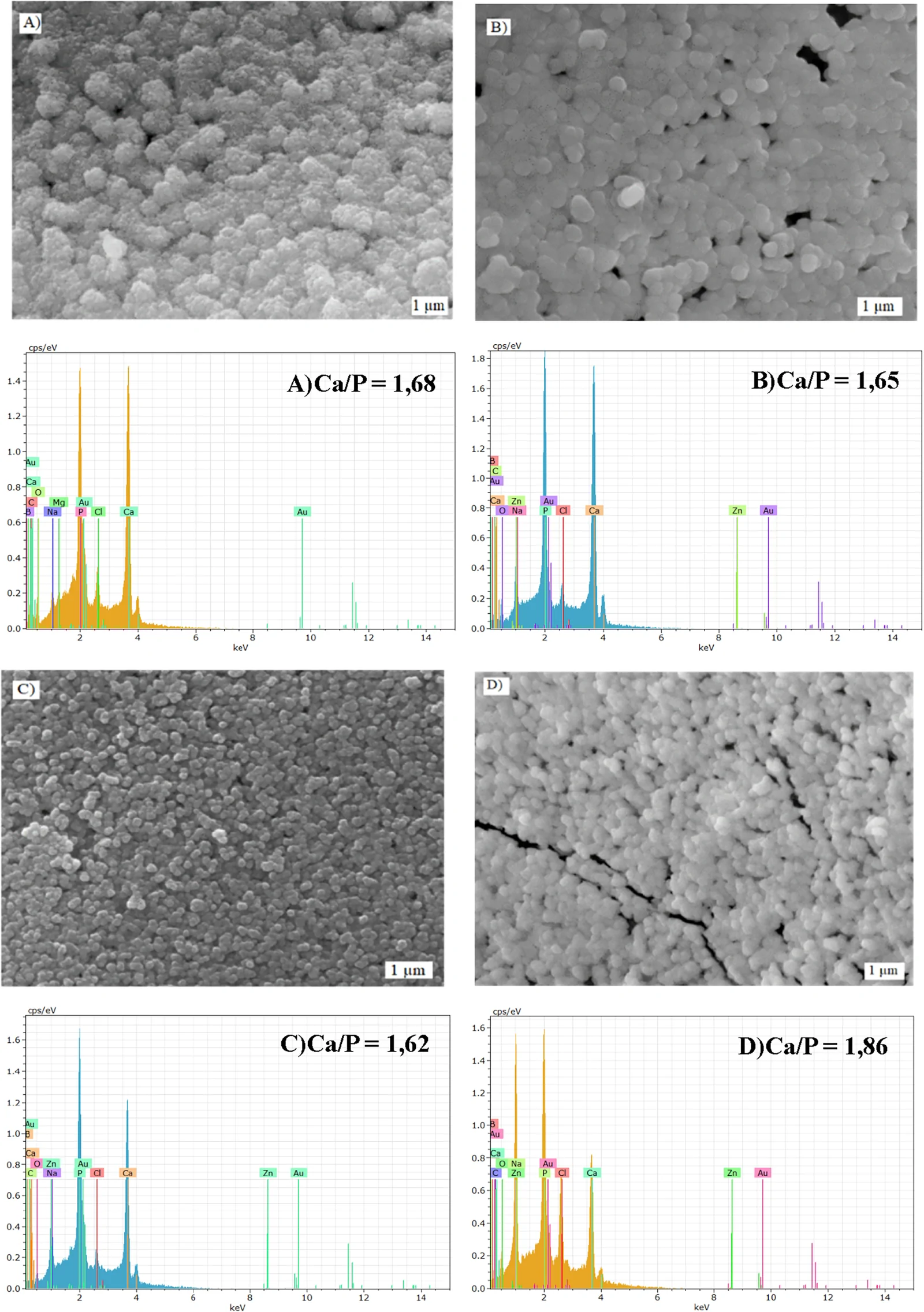

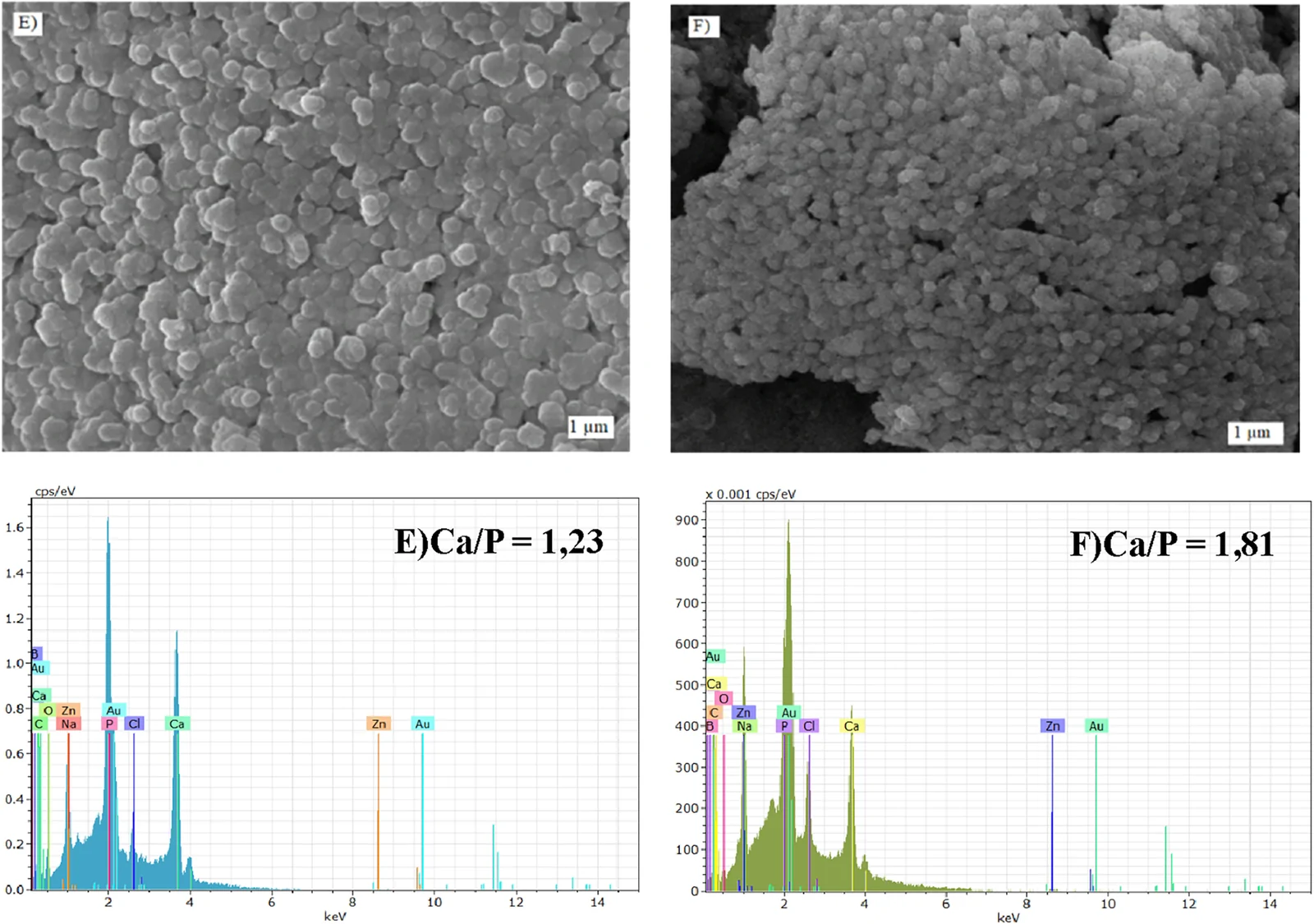
SEM micrography and EDS spectra of CaNaB (A); 1ZnO (B); 2.5ZnO (C), 5ZnO (D), 7.5ZnO (E), and 10ZnO (F) samples after immersion in SBF for 21 days. Ca/P atomic ratio are included for all the samples.

All glasses presented boron (B), calcium (Ca), phosphorus (P), gold (Au), oxygen (O), sodium (Na), carbon (C), and silver (Ag). Gold (Au) peaks in the spectra resulted from the surface metallization required for sample conductivity during. SEM images revealed the presence of spherical structures on the surface of all samples, which are typical of HAp formation in *in vitro* tests using SBF.^69,70^ The Ca/P atomic ratios were calculated from the EDS data and are presented in Figure 15 and Table 3, with values ranging from 1.23 to 1.86. The Ca/P ratio of stoichiometric HAp, used for bone implants, is 1.67.^67^ Thus, the Ca/P ratios of the CaNaB, 1ZnO, and 2.5ZnO samples (1.68, 1.65, and 1.62, respectively) indicate the formation of HAp or amorphous calcium phosphate on the surface of the glass samples.

**Table 3:** Ca/P Ratio of Vitreous Samples and Stoichiometric HA for Comparison

Samples and HA	Ca/P
CaNaB	1.68
1ZnO	1.65
2.5ZnO	1.62
5ZnO	1.86
7.5ZnO	1.23
10ZnO	1.81
Stoichiometric hydroxyapatite	1.67 ^72^

However, although the SEM images of the 1ZnO, 7.5ZnO, and 10ZnO samples revealed the formation of spherical apatite-like structures on their surfaces, the diffractograms of these glasses (Section 3.9) did not show peaks corresponding to crystalline hydroxyapatite. As discussed in Section 3.9, during the immersion of the glasses in SBF, an amorphous calcium phosphate layer forms initially; this layer exhibits a morphology similar to that of HA but does not exhibit peaks in the diffractograms.^56,71^

Furthermore, as also reported in previous discussions, the incorporation of higher ZnO concentrations increases the chemical durability of the glass network and, consequently, retards apatite crystallization. Therefore, the presence of spherical structures in the SEM images of the 1ZnO, 7.5ZnO, and 10ZnO samples is likely due to the presence of an amorphous calcium phosphate layer.

### 3.12 Cell Death and Cell Scratch Migration Assays

A trypan blue exclusion assay was performed to evaluate the potential cytotoxicity of the bioactive glasses on human cells. As the (Figure 16a) shows, the exposure of human fibroblasts to either the CaNaB or the 10ZnO bioactive formulation at 500 μg/mL do not elicit a statistically significant increase in cell death relative to untreated controls. The absence of significant cytotoxic effects at this high concentration underscores the materials’ biocompatibility and provides basis for its subsequent uses in more sophisticated *in vitro* and *in vivo* tests. Representative images (Figure 16b) further substantiate this interpretation, revealing intact cell morphology and preserved confluence. Together, these data support the safety of the studied bioactive glasses for human cells and justify subsequent investigations in animal models and, ultimately, translational evaluation in clinical settings.

**Figure 16:**
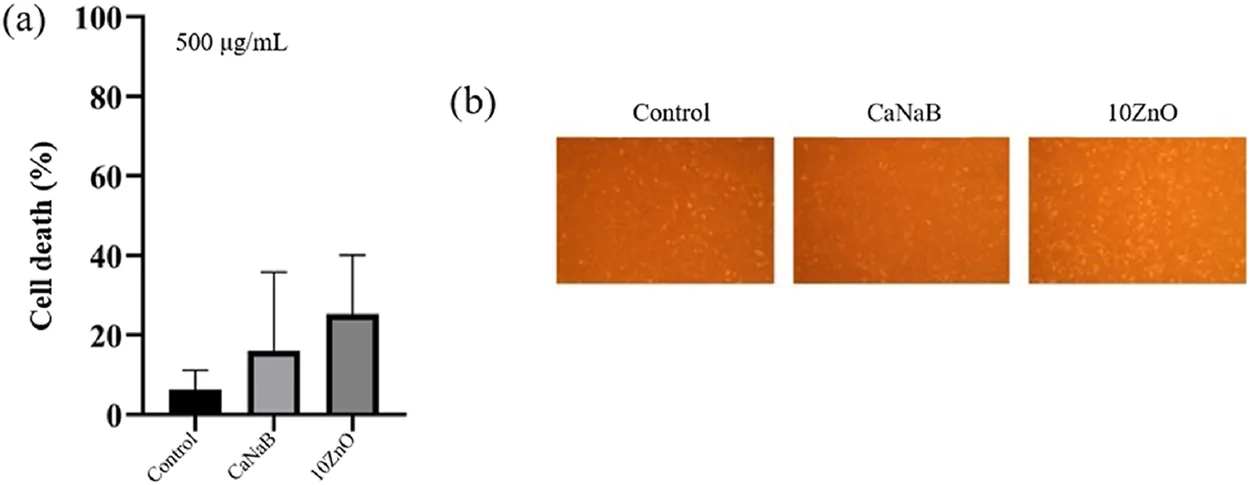
Cell death analysis by trypan blue exclusion (a and b).

In the wound-healing assay, the quantitative migration analysis (Figure 17a) shows that neither the CaNaB nor the glass compositions containing 1%, 5%, or 10% of the ZnO impaired fibroblast motility over the experimental period. Migration rates across all treatment groups remained comparable to those of the control. Representative images captured at 0 and 24 h (Figure 17b) demonstrate a consistent advance of the migratory front in all conditions and no detectable alterations in cell morphology or wound-closure dynamics attributable to the materials. Collectively, these results indicate that the bioactive glasses preserve the intrinsic migratory capacity of human fibroblasts, further reinforcing their biocompatibility and supporting their progression into *in vivo* animal models, where the 3D and more complex microenvironment would be more subsequent translational studies.

**Figure 17:**
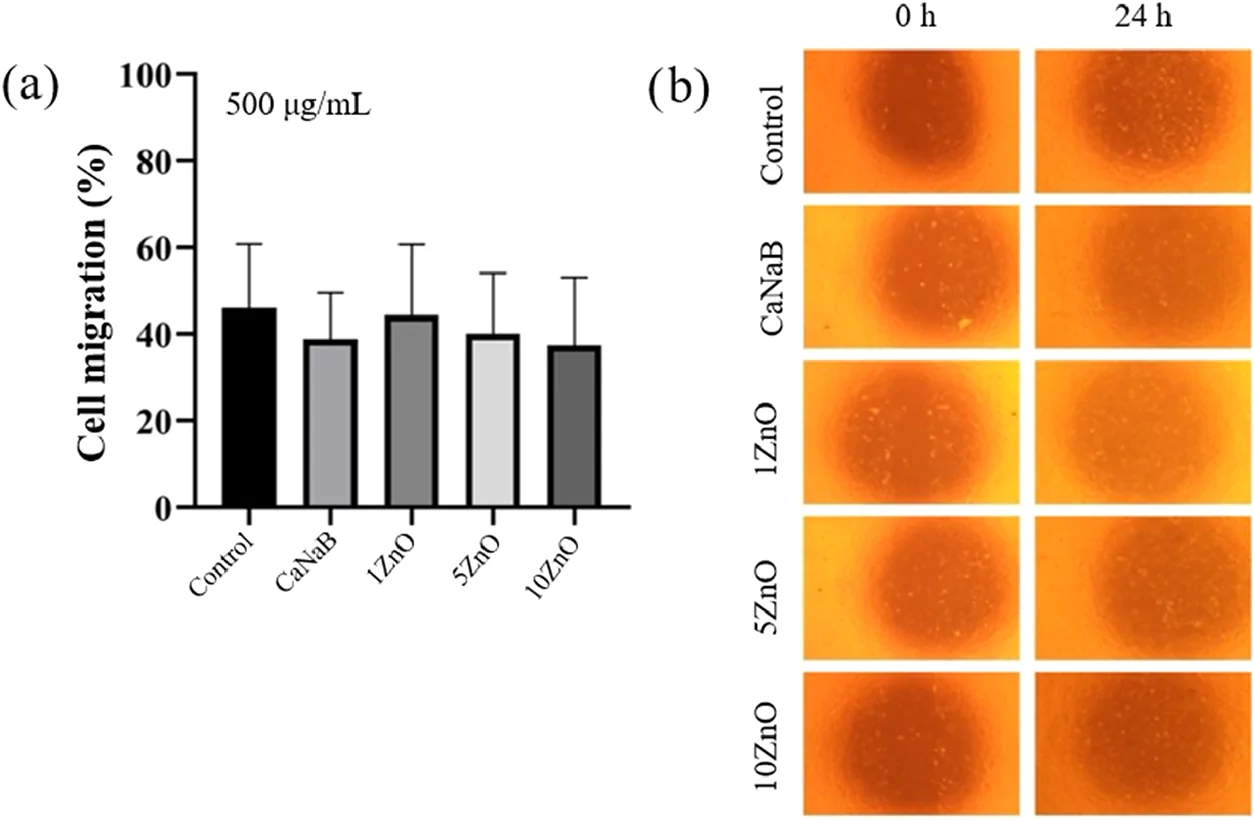
Cell migration assessment by the scratch assay (a and b).

The use of Zn-dopped materials could contribute to the wound healing process *in vivo* as it has been already shown that healing-rates is nearly three times greater in patients which received oral supplementation with zinc sulfate compared to nonsupplemented ones.^73^ In fact, Zn plays a critical role in numerous physiological processes, including development, immune function, and wound healing, and impairments in wound repair can be reversed through Zn supplementation.^73–75^

In the molecular level, it has been shown that a wide range of metalloenzymes and transcription factors depend on Zn for their proper structural integrity and functional activity.^76^ Furthermore, by using genetically engineered mice, it was already reported that Zn is a pro-inflammatory signal and that the Zn/GPR39/IL-6 axis plays a critical role in wound healing process. These authors showed that ZnT2, a Zn transporter, is important for the release of Zn by mast cells, which are key components of the immune system during this inflammatory process. In this context, the use of Zn-dopped biomaterials, as the one reported here, could implicate in the improvement of the first phase of wound healing process.^77^

### 3.13 Antibacterial Activity Analysis

The antibacterial activity of the glass samples was tested against the Gram-positive bacteria *S. aureus* and the Gram-negative bacteria *E. coli*. Figure 18 shows photographs of the test plates containing the Gen 10 antibiotic disk (gentamicin 10 μg) and glass samples discs after 24 h of testing against the bacteria *S. aureus*.

**Figure 18:**
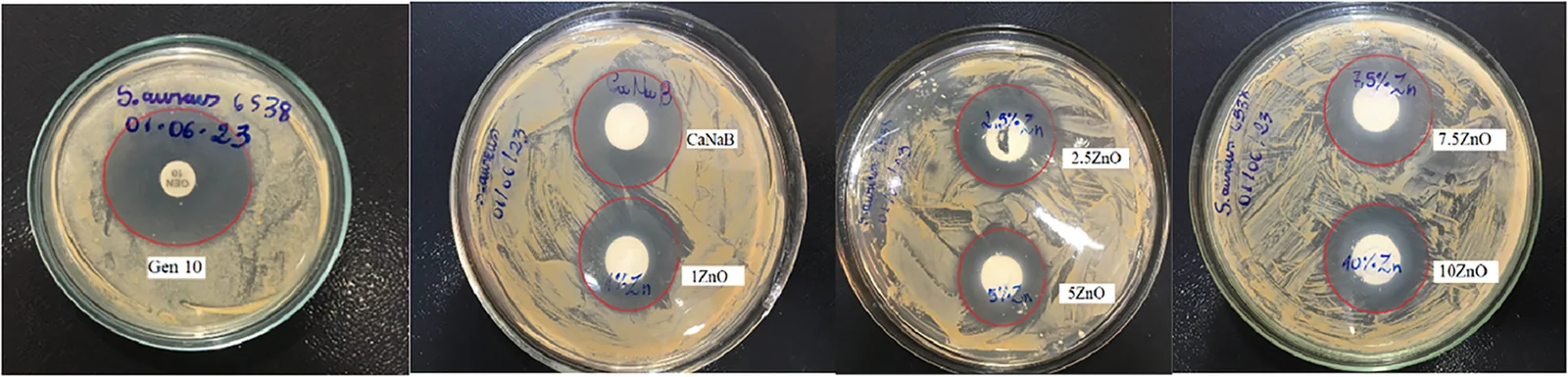
Photographs of the plates containing the antibiotic Gen gentamicin and the glass samples tested for 24 h against the bacteria *S. aureus*. The red circle demarcates the zone of bacterial growth inhibition.

Observing Figure 18, it is possible to indicate that the glass samples CaNaB, 1ZnO, 2.5ZnO, 5ZnO, 7.5ZnO, and 10ZnO, as well as the antibiotic, showed antibacterial effect against the bacteria *S. aureus*. Because the halo formed within the red circle corresponds to the zone of inhibition of bacterial growth, its formation is considered proof of the material’s antibacterial action.^78^

Figure 19 shows of the antibiotic and vitreous samples tested against *E. coli* bacteria after 24 h of incubation.

**Figure 19:**
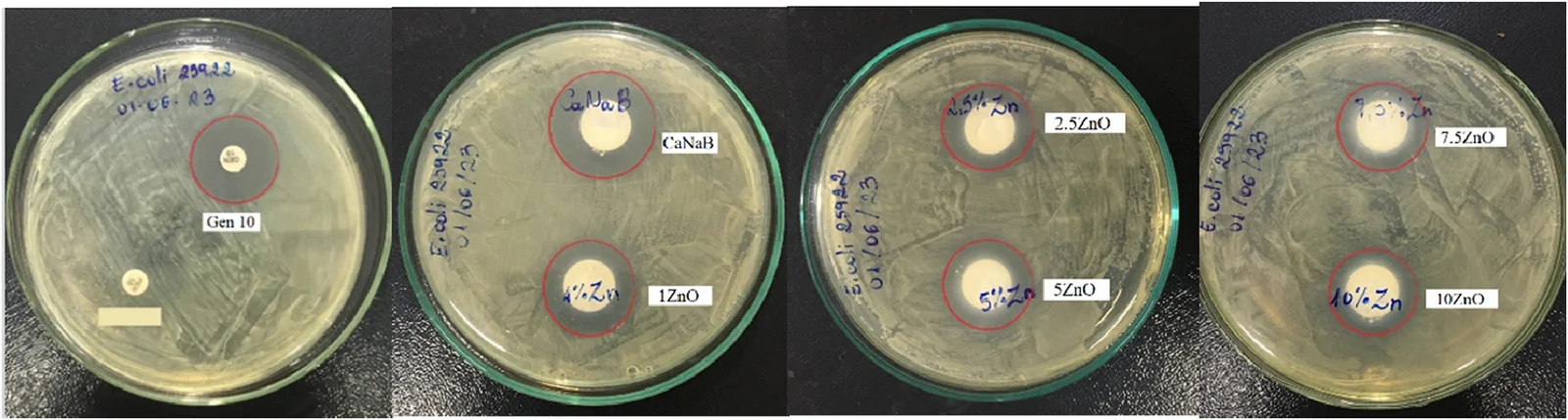
Photographs of the plates containing the antibiotic gentamicin and the glass samples tested for 24 h against the bacteria *E. coli*. The red circle demarcates the area of bacterial growth inhibition.

Figure 19 indicates that the vitreous samples and the antibiotic Gen 10 also showed antibacterial activity against the *E. coli* bacteria, as a zone of bacterial growth inhibition around the samples is notable. Table 4 presents the diameters of the bacterial growth inhibition zones that each vitreous sample and antibiotic caused when tested against the bacteria *S. aureus* and *E. coli* for 24 h.

**Table 4:** Antibiotic and Glass Samples Tested and Their Respective Diameters of the Bacterial Growth Inhibition Zone, in Millimeters, against the Bacteria *S. aureus* and *E. coli*

	Inhibition zone diameter (mm)	
Antibiotic and glass samples	*S. aureus*	*E. coli*
Gen 10	17.2	12
CaNaB	17.8	15.17
1ZnO	11.86	11.57
2.5ZnO	15.07	8.95
5ZnO	13.55	9
7.5ZnO	13.25	9.25
10ZnO	13.35	9.7

Based on the statistical analysis performed (one-way ANOVA with a significance level of 0.05, 95% confidence interval, *F*-value of 1.1895, and *p*-value of 0.4073), it was found that, in the assay against *S. aureus*, the mean inhibition zone diameters for all glass samples did not show significant differences. This suggests that the ZnO incorporation into the glass network did not significantly affect the antibacterial effect of the material.

Babu et al. stated that antibacterial activity is related to the material composition, dissolution rate, release of antibacterial ions, and changes in pH values.^79^ The lack of improvement in antibacterial activity upon the addition of zinc – despite its known antibacterial properties,^9,13,18,21,22^ was likely due to the increased chemical stability and reduced dissolution rate resulting from the higher concentration of ZnO in the glass network. This reduces the release of antibacterial ions from the material into the environment.^79,80^ This behavior is also indicated by the mass loss results and pH measurements.

Elmowafy et al. obtained similar results when testing copper- and zinc-doped borate glasses against *S. aureus* and *E. coli* bacteria. Using the disk diffusion method, it was observed that antibacterial activity did not vary significantly with the concentration of added zinc and copper, elements described in the literature as antibacterial.^81^

Based on a one-way ANOVA statistical analysis (significance level of 0.05, 95% confidence interval, *F*-value of 10.5957, and *p*-value of 0.00324) performed on the results of the assay against *E. coli*, it was concluded that there is a significant difference among the samples. Pairwise comparison using Tukey’s test revealed that, in tests involving *E. coli* bacteria, samples CaNaB and 1ZnO exhibited the largest inhibition zone diameters, with no significant difference between them. Samples 2.5ZnO, 5ZnO, 7.5ZnO, and 10ZnO showed lower values than sample CaNaB but did not differ significantly from one another. This can also be explained due to the lower release of antibacterial ions due to the greater stability of the glass network, and less dissolution of the material due to the greater zinc incorporation, as previously mentioned in this section.^80^

In the antibacterial assay against *S. aureus* bacteria, there was no significant difference between the inhibition zones of the glasses and the antibiotic gentamicin. In the test with *E. coli* bacteria, none of the vitreous samples showed a significant difference with the Gen 10 control. Therefore, all of the vitreous samples showed antibacterial activity comparable to that of the antibiotic tested.

## 4 Conclusion

In this study, calcium–sodium-borate glasses with ZnO were produced in concentrations of 0, 1, 2.5, 5, 7.5, and 10 mol % to investigate the influence of ZnO on bioactivity and other relevant properties of the material. An increase in density and N_4_ fraction and a decrease in molar volume with the replacement of B_2_O_3_ by ZnO indicate that the ZnO addition modified the material structure. The DTA analysis revealed an increase in *T*_x_ and thermal stability with the ZnO addition, which represents an increase in the working temperature range of this glass material, beneficial for applications such as thermally molded devices. The radiopacity increased with the ZnO insertion and the samples 5ZnO, 7.5ZnO, and 10ZnO exhibited radiopacity higher than that of dentin. Macrophage cell viability assay showed that all samples were noncytotoxic.

The FTIR spectra, XRD patterns, SEM micrographs, and EDS spectra of the samples, obtained after immersion in SBF, indicated the bioactivity of the glasses, as evidenced by the formation of hydroxyapatite layer in the CaNaB, 1ZnO, 2.5ZnO, and 5ZnO samples. Due to the higher concentration of ZnO, the 7.5ZnO and 10ZnO samples did not show evidence of HA formation during the periods of immersion in SBF investigated, but it cannot be excluded that these glasses may exhibit bioactivity of these glass samples after longer periods of immersion.

Based on the results of the mass loss, pH, FTIR, and XRD analyses, it is possible to conclude that higher ZnO concentrations increased the chemical stability of the material, thereby reducing the degradation rate and delaying the HA formation rate of the glasses during immersion in SBF.

Cell death and scratch cell migration assays showed that the addition of ZnO to the glasses did not significantly increase the percentage of cell death and preserved the intrinsic migratory capacity of human fibroblasts, indicating their potential for wound healing applications.

All vitreous samples showed antibacterial properties against the Gram-positive bacteria *S. aureus* and the Gram-negative bacteria *E. coli*. In general, the samples showed relevant potential for applications in bone tissue repair and the 5ZnO sample exhibited the most favorable combination of properties: high thermal stability, radiopacity, antibacterial activity, and bioactivity.

## References

[1] Zhao S., Li L., Wang H., Zhang Y., Cheng X., Zhou N., Rahaman M. N., Liu Z., Huang W., Zhang C. (2015). Wound dressings
composed of copper-doped borate bioactive glass microfibers stimulate
angiogenesis and heal full-thickness skin defects in a rodent model. Biomaterials.

[2] Yin H., Yang C., Gao Y., Wang C., Li M., Guo H., Tong Q. (2018). Fabrication
and characterization of strontium-doped
borate-based bioactive glass scaffolds for bone tissue engineering. J. Alloys Compd..

[3] Cui X., Huang W., Zhou J., Wang H., Zhou N., Wang D., Cui X., Huang C., Yu Z., Wang L., Liu W., Wang T., Pan H., Zhang Y., Rahaman M. N., Rahaman M. N. (2017). Evaluation of an
injectable bioactive borate glass cement to heal bone defects in a
rabbit femoral condyle model. Mater. Sci. Eng.
C.

[4] Cui X., Zhang Y., Wang H., Gu Y., Li L., Zhou J., Zhao S., Huang W., Zhou N., Wang D., Pan H., Rahaman M. N. (2016). An injectable
borate
bioactive glass cement for bone repair: Preparation, bioactivity and
setting mechanism. J. Non-Cryst. Solids.

[5] Leon-Villapalos J., Eldardiri M., Dziewulski P. (2010). The use of human deceased donor skin
allograft in burn care. Cell Tissue Bank.

[6] Hench L. L., Splinter R. J., Allen W. C., Greenlee T. K. (1971). Bonding Mechanisms
at the Interface of Ceramic Prosthetic Materials. J. Biomed. Mater. Res..

[7] Hench L. L. (2006). The story
of Bioglass. J. Mater. Sci. Mater. Med..

[8] Jones J. R., Brauer D. S., Hupa L., Greenspan D. C. (2016). Bioglass
and Bioactive Glasses and Their Impact on Healthcare. Int. J. Appl. Glass Sci..

[9] Schuhladen K., Wang X., Hupa L., Boccaccini A. R. (2018). Dissolution
of borate and borosilicate bioactive glasses and the influence of
ion (Zn, Cu) doping in different solutions. J. Non-Cryst. Solids.

[10] MacDonald K., Hanson M. A., Boyd D. (2016). Modulation
of strontium release from
a tertiary borate glass through substitution of alkali for alkali
earth oxide. J. Non-Cryst. Solids.

[11] Abdelghany A. M., Kamal H. (2014). Spectroscopic investigation
of synergetic bioactivity behavior of
some ternary borate glasses containing fluoride anions. Ceram. Int..

[12] Baino F. (2018). Bioactive
glasses – When glass science and technology meet regenerative
medicine. Ceram. Int..

[13] Mutlu N., Kurtuldu F., Unalan I., Neščáková Z., Kaňková H., Galusková D., Michálek M., Liverani L., Galusek D., Boccaccini A. R. (2022). Effect
of Zn and Ga doping on bioactivity, degradation, and antibacterial
properties of borate 1393-B3 bioactive glass. Ceram. Int..

[14] Yao A., Wang D., Huang W., Fu Q., Rahaman M. N., Day D. E. (2007). In vitro
bioactive characteristics of borate-based
glasses with controllable degradation behavior. J. Am. Ceram. Soc..

[15] Pan H. B., Zhao X. L., Zhang X., Zhang K. B., Li L. C., Li Z. Y., Lam W. M., Lu W. W., Wang D. P., Huang W. H., Lin K. L., Chang J. (2010). Strontium borate glass:
Potential biomaterial for bone regeneration. J. R. Soc. Interface.

[16] Fu H., Fu Q., Zhou N., Huang W., Rahaman M. N., Wang D., Liu X. (2009). In vitro evaluation of borate-based bioactive glass scaffolds prepared
by a polymer foam replication method. Mater.
Sci. Eng. C.

[17] Kaur G., Pandey O. P., Singh K., Homa D., Scott B., Pickrell G. (2014). A review of bioactive glasses: Their structure, properties,
fabrication and apatite formation. J. Biomed.
Mater. Res., Part A.

[18] Yazdi A. R., Towler M. (2016). The effect of the addition of gallium on the structure
of zinc borate glass with controlled gallium ion release. Mater. Des..

[19] Lepry W. C., Smith S., Nazhat S. N. (2018). Effect of sodium
on bioactive sol-gel-derived
borate glasses. J. Non-Cryst. Solids.

[20] Ghazy A. R., Elmowafy B. M., Abdelghany A. M., Meaz T. M., Ghazy R., Ramadan R. M. (2023). Structural, optical,
and cytotoxicity studies of laser
irradiated ZnO doped borate bioactive glasses. Sci. Rep..

[21] Kaya S., Cresswell M., Boccaccini A. R. (2018). Materials Science & Engineering
C Mesoporous silica-based bioactive glasses for antibiotic-free antibacterial
applications. Mater. Sci. Eng., C.

[22] Kermani F., Nazarnezhad S., Mollaei Z., Mollazadeh S., Ebrahimzadeh-Bideskan A., Askari V. R., Oskuee R. K., Moradi A., Hosseini S. A., Azari Z., Baino F., Kargozar S. (2023). Zinc- and Copper-Doped
Mesoporous Borate Bioactive
Glasses: Promising Additives for Potential Use in Skin Wound Healing
Applications. Int. J. Mol. Sci..

[23] Mehrabi T., Mesgar A. S. (2022). In vitro biomineralization
potential in simulated wound
fluid and antibacterial efficacy of biologically-active glass nanoparticles
containing B2O3/ZnO. Colloids Surf., B.

[24] Yamaguchi M. (1998). Role of Zinc
in Bone Formation and Bone Resorption. J. Trace
Elem. Exp. Med..

[25] Brauer D. S., Al-Noaman A., Hill R. G., Doweidar H. (2011). Density-structure correlations
in fluoride-containing bioactive glasses. Mater.
Chem. Phys..

[26] Mosmann T. (1983). Rapid Colorimetric
Assay for Cellular Growth and Survival : Application to Proliferation
and Cytotoxicity Assays. J. Immunol. Methods.

[27] Kokubo T., Kushitani H., Sakka S., Kitsugi T., Yamamuro T. (1990). Solutions
able to reproduce in vivo surface-structure changes in bioactive glass-ceramic
A-W3. J. Non-Cryst. Solids.

[28] Manupriya K. S., Thind G., Sharma K., Singh V., Rajendran S., Aravindan (2007). Soluble borate
glasses: In vitro analysis. J. Am. Ceram. Soc..

[29] Huang W., Day D. E., Kittiratanapiboon K., Rahaman M. N. (2006). Kinetics and mechanisms
of the conversion of silicate (45S5), borate, and borosilicate glasses
to hydroxyapatite in dilute phosphate solutions. J. Mater. Sci. Mater. Med..

[30] Othman H., Valiev D., Polisadova E. (2017). Structural
and mechanical properties
of zinc aluminoborate glasses with different content of aluminum oxide. J. Mater. Sci.:Mater. Electron..

[31] da
Silva G. L., Rodrigues I. F., Pereira S. S. S., Fontoura G. M. G., Reis A. S., Pedrochi F., Steimacher A. (2022). Bioactive
antibacterial borate glass and glass-ceramics. J. Non-Cryst. Solids.

[32] Crowley L. C., Marfell B. J., Christensen M. E., Waterhouse N. J. (2016). Measuring
cell death by trypan blue uptake and light microscopy. Cold Spring Harbor Protoc..

[33] Topper B., Mo D., Youngman R. E., Varsamis C. P. E. (2023). Zinc borate glasses:
properties, structure and modelling of the composition-dependence
of borate speciation. Phys. Chem. Chem. Phys..

[34] Ahmad F., Hassan Aly E., Atef M., Elokr M. M. (2014). Study the influence
of zinc oxide addition on cobalt doped alkaline earth borate glasses. J. Alloys Compd..

[35] Kaur M., Singh S. P., Mudahar D. S., Mudahar G. S. (2012). Structural
investigation
of B2O3-Li2 CO3-Al2O3 glasses by molar volume measuremenmts and FTIR
spectroscopy. Mater. Phys. Mech..

[36] O’Connell K., Hanson M., O’Shea H., Boyd D. (2015). Linear release of strontium
ions from high borate glasses via lanthanide/alkali substitutions. J. Non. Cryst. Solids.

[37] Salman S. M., Salama S. N., Abo-Mosallam H. A. (2012). The role
of strontium and potassium
on crystallization and bioactivity of Na2O-CaO-P2O5-SiO2glasses. Ceram. Int..

[38] Abdelghany A. M., Ouis M. A., Azooz M. A., Elbatal H. A., El-Bassyouni G. T. (2016). Role of
SrO on the bioactivity behavior of some ternary borate glasses and
their glass ceramic derivatives. Spectrochim.
Acta A Mol. Biomol. Spectrosc..

[39] Deliormanlı A. M. (2013). Size-dependent
degradation and bioactivity of borate bioactive glass. Ceram. Int..

[40] Clupper D. C., Mecholsky J. J., LaTorre G. P., Greenspan D. C. (2002). Bioactivity
of tape cast and sintered bioactive glass-ceramic in simulated body
fluid. Biomaterials.

[41] Bi L., Rahaman M. N., Day D. E., Brown Z., Samujh C., Liu X., Mohammadkhah A., Dusevich V., Eick J. D., Bonewald L. F. (2013). Acta Biomaterialia
Effect of bioactive borate glass microstructure on bone regeneration,
angiogenesis, and hydroxyapatite conversion in a rat calvarial defect
model. Acta Biomater..

[42] Moustafa Y., Doweidar H. (1994). Utilisation of Infrared
Spectroscopy to Determine the
Fraction of the Four Coordinated Borons in Borate Glasses. Phys. Chem. Glasses.

[43] ISO 10993 . Biological evaluationof medical devices. Part 5: Tests for in vitro cytotoxicity International Standard ISO 10993–5 2009.

[44] Kullberg A. T. G., Lopes A. A. S., Veiga J. P. B., Lima M. M. R. A., Monteiro R. C. C. (2016). Formation and crystallization of zinc borosilicate
glasses: Influence of the ZnO/B2O3 ratio. J.
Non-Cryst. Solids.

[45] Naresh P., Padmaja A., Kumar K. S. (2020). Influence
of zinc oxide addition
on the biological activity and electrical transport properties of
TeO2–Li2O–B2O3 glasses. Materialia.

[46] Kehoe S., Tonkopi E., Abraham R. J., Boyd D. (2012). Predicting the thermal
responses and radiopacity of multicomponent zinc-silicate bioglasses:
A focus on ZnO, La 2O 3, SiO 2 and TiO 2. J.
Non. Cryst. Solids.

[47] Chiang T. Y., Lu Y. C., Chen C. C., Ding S. J. (2025). Enhancing Radiopacity
and Antibacterial Activity of Osteogenic Calcium Silicate Cement by
Incorporating Strontium. J. Funct. Biomater..

[48] Vivan R. R., Ordinola-Zapata R., Bramante C. M., Bernardineli N., Garcia R. B., Hungaro Duarte M. A., de Moraes I. G. (2009). Evaluation
of the radiopacity of some commercial and experimental root-end filling
materials. Oral Surg., Oral Med., Oral Pathol.,
Oral Radiol., Endodontol..

[49] Wajda A., Goldmann W. H., Detsch R., Boccaccini A. R., Sitarz M. (2019). Influence of zinc ions on structure, bioactivity, biocompatibility
and antibacterial potential of melt-derived and gel-derived glasses
from CaO-SiO 2 system. J. Non-Cryst. Solids.

[50] Kaur G., Pickrell G., Kimsawatde G., Homa D., Allbee H. A., Sriranganathan N. (2014). Synthesis,
cytotoxicity, and hydroxyapatite formation
in 27-Tris-SBF for sol-gel based CaO-P2O5-SiO2-B2O 3-ZnO bioactive
glasses. Sci. Rep..

[51] da
Silva L. C. A., Neto F. G., Pimentel S. S. C., da R., Palácios S., Sato F., Retamiro K. M., Fernandes N. S., Nakamura C. V., Pedrochi F., Steimacher A. (2021). The role of
Ag2O on antibacterial and bioactive properties of borate glasses. J. Non-Cryst. Solids.

[52] Ansari A., Moztarzadeh F., Bizari D., Ashuri M., Tahriri M. (2012). Ion release
behavior and apatite-forming ability of sol-gel derived 70S30C bioactive
glass with magnesium/ zinc substitution. Key
Eng. Mater..

[53] Babu M. M., Rao P. V., Singh R. K., Kim H. W., Veeraiah N., Özcan M., Prasad P. S. (2021). ZnO incorporated high phosphate bioactive
glasses for guided bone regeneration implants: enhancement of in vitro
bioactivity and antibacterial activity. J. Mater.
Res. Technol..

[54] Balamurugan A., Balossier G., Kannan S., Michel J., Rebelo A. H. S., Ferreira J. M. F. (2007). Development and in vitro characterization
of sol-gel derived CaO-P2O5-SiO2-ZnO bioglass. Acta Biomater..

[55] Bejarano J., Caviedes P., Palza H. (2015). Sol-gel synthesis and
in vitro bioactivity
of copper and zinc-doped silicate bioactive glasses and glass-ceramics. Biomedical Materials.

[56] Hench L. L. (1991). Bioceramics:
from concept to clinic. J Am Ceram Soc. 1993;72:93–98. J. Am. Ceram. Soc..

[57] Aina V., Malavasi G., Fiorio Pla A., Munaron L., Morterra C. (2009). Zinc-containing
bioactive glasses: Surface reactivity and behaviour towards endothelial
cells. Acta Biomater..

[58] Chen J., Zeng L., Chen X., Liao T., Zheng J. (2018). Preparation
and characterization of bioactive glass tablets and evaluation of
bioactivity and cytotoxicity in vitro. Bioact.
Mater..

[59] Samudrala R. K., Patel S., Penugurthi V., Manavathi B., A A. P. (2021). In vitro studies of B2O3–SiO2–Na2O–CaO–ZnO
bioactive glass system. J. Non-Cryst. Solids.

[60] Han X., Day D. E. (2007). Reaction of sodium
calcium borate glasses to form hydroxyapatite. J. Mater. Sci. Mater. Med..

[61] Guillen-Romero L. D., Oropeza-Guzmán M. T., López-Maldonado E. A., Iglesias A. L., Paz-González J.
A., Ng T., Serena-Gómez E., Villarreal-Gómez L. J. (2019). Synthetic hydroxyapatite
and its use in bioactive coatings. J. Appl.
Biomater. Funct. Mater..

[62] Phatai P., Futalan C. M., Utara S., Khemthong P., Kamonwannasit S. (2018). Structural characterization of cerium-doped
hydroxyapatite
nanoparticles synthesized by an ultrasonic-assisted sol-gel technique. Results Phys..

[63] Klee W. E., Engel G. (1970). I.R. spectra of the phosphate ions
in various apatites. J. Inorg. Nucl. Chem..

[64] Koutsopoulos S. (2002). Synthesis
and characterization of hydroxyapatite crystals : A review
study on the analytical methods. J. Biomed.
Mater. Res..

[65] Fowler B. (1974). Infrared Studies
of Apatites. 1. Vibrational Assignments for Calcium, Strontium, and
Barium Hydroxyapatites Utilizing Isotopic-Substitution. Inorg. Chem..

[66] Neščáková Z., Zheng K., Liverani L., Nawaz Q., Galusková D., Kaňková H., Michálek M., Galusek D., Boccaccini A. R. (2019). Multifunctional zinc ion doped sol
– gel derived mesoporous bioactive glass nanoparticles for
biomedical applications. Bioact. Mater..

[67] Huang W., Day D. E., Kittiratanapiboon K., Rahaman M. N. (2006). Kinetics and Mechanisms
of the conversion of silicate (45S5), borate, and borosilicate glasses
to hydroxyapatite in dilute phosphate solutions. J. Mater. Sci. Mater. Med..

[68] Montazeri N., Jahandideh R., Biazar E. (2011). Synthesis of fluorapatite-hydroxyapatite
nanoparticles and toxicity investigations. Int.
J. Nanomed..

[69] Queiroz M. N., Morais T. S., Dias T. G., Barros J. D. S., Dutra R. P., Reis A. S., de Menezes A. S., Steimacher A., Pedrochi F. (2024). Magnesium borate-glasses for biomedical
application:
Physicochemical and in vitro bioactive properties, antibacterial activity
and cell viability. J. Non-Cryst. Solids.

[70] Fu Q., Rahaman M. N., Fu H., Liu X. (2010). Silicate, borosilicate,
and borate bioactive glass scaffolds with controllable degradation
rate for bone tissue engineering applications. I. Preparation and
in vitro degradation. J. Biomed. Mater. Res.,
Part A.

[71] Filgueiras M. R. T., La Torre G., Hench L. L. (1993). Solution
effects on the surface reactions
of three bioactive glass compositions. J. Biomed.
Mater. Res..

[72] Liu H., Yazici H., Ergun C., Webster T. J., Bermek H. (2008). An in vitro
evaluation of the Ca/P ratio for the cytocompatibility of nano-to-micron
particulate calcium phosphates for bone regeneration. Acta Biomater..

[73] Pories W. J., Henzel J. H., Rob C. (1967). Acceleration
of wound
healing in man with zinc sulphate given by mouth. Lancet.

[74] Fukada T., Yamasaki S., Nishida K., Murakami M., Hirano T. (2011). Zinc homeostasis
and signaling in health and diseases. JBIC,
J. Biol. Inorg. Chem..

[75] Lin P. H., Sermersheim M., Li H., Lee P. H. U., Steinberg S. M., Ma J. (2018). Zinc in wound healing modulation. Nutrients.

[76] Andreini C., Banci L., Bertini I., Rosato A. (2006). Counting the zinc-proteins
encoded in the human genome. J. Proteome Res..

[77] Nishida K., Hasegawa A., Yamasaki S., Uchida R., Ohashi W., Kurashima Y., Kunisawa J., Kimura S., Iwanaga T., Watarai H., Hase K., Ogura H., Nakayama M., Kashiwakura J. ichi., Okayama Y., Kubo M., Ohara O., Kiyono H., Koseki H., Murakami M., Hirano T. (2019). Mast cells
play role in wound healing through the ZnT2/GPR39/IL-6 axis. Sci. Rep..

[78] Naruphontjirakul P., Kanchanadumkerng P., Ruenraroengsak P. (2023). Multifunctional Zn and Ag co-doped
bioactive glass nanoparticles for bone therapeutic and regeneration. Sci. Rep..

[79] Babu M. M., Bhemarajam J., Lakshmi A. M., Murimadugula S., Devi T. I., Sroda M., Özcan M., Rao P. V., Prasad P. S. (2023). Exploring the Potential of Silica
Mixed Zinc Phosphate Bioactive Glasses for Bone Regeneration: In Vitro
Bioactivity and Antibacterial Activity Analysis. J. Non-Cryst. Solids.

[80] Hameed A. S., Muhammed A., Luma A., Hamood J., Obaid I. A. (2015). Effect
of Zinc on Antibacterial Action of Bioactive Glass Coating for Dental
Implant. Med. J. Babylon.

[81] Elmowafy B. M., Ramadan R. M., Ghazy A. R., Fahim I. S., Meaz T. M., Ghazy R., Abdelghany A. M. (2025). Effect
of copper and zinc oxide doping
in 13–93B3 borate glass for enhanced wound healing. Ceram. Int..

